# PPARδ Orchestrates a Prometastatic Metabolic Response to Microenvironmental Cues in Pancreatic Cancer

**DOI:** 10.1158/0008-5472.CAN-24-3475

**Published:** 2025-07-03

**Authors:** Beatriz Parejo-Alonso, David Barneda, Sara Maria David Trabulo, Sarah Courtois, Sara Compte-Sancerni, Jelena Zurkovic, Laura Ruiz-Cañas, Quan Zheng, Jiajia Tang, Matthias M. Gaida, Ulf Schmitz, Pilar Irun, Laure Penin-Peyta, Shanthini Mary Crusz, Petra Jagušt, Pilar Espiau-Romera, Alba Royo-García, Andrés Gordo-Ortiz, Mariia Yuneva, Meng-Lay Lin, Shenghui Huang, Ming-Hsin Yang, Angel Lanas, Bruno Sainz, Christoph Thiele, Christopher Heeschen, Patricia Sancho

**Affiliations:** 1 Aragon Health Research Institute (IIS Aragón), Zaragoza, Spain.; 2 Barts Cancer Institute, Queen Mary University of London, London, United Kingdom.; 3 LIMES Life and Medical Sciences Institute, University of Bonn, Bonn, Germany.; 4 Department of Biochemistry, Autónoma University of Madrid (UAM), School of Medicine, Instituto de Investigaciones Biomédicas (IIBm) Sols-Morreale (CSIC-UAM), Madrid, Spain.; 5 Cancer, Area 3, Instituto Ramón y Cajal de Investigación Sanitaria (IRYCIS), Madrid, Spain.; 6 Center for Single-Cell Omics and State Key Laboratory of Systems Medicine for Cancer, Shanghai Jiao Tong University School of Medicine, Shanghai, China.; 7 Institute of Pathology, University Medical Center Mainz, JGU-Mainz, Mainz, Germany.; 8 TRON Translational Oncology at the University Medical Center, JGU-Mainz, Mainz, Germany.; 9 Research Center for Immunotherapy, University Medical Center Mainz, JGU-Mainz, Mainz, Germany.; 10 Computational Biomedicine Lab, College of Science and Engineering, James Cook University, Townsville, Australia.; 11 Liver and Digestive Diseases Networking Biomedical Research Centre (CIBEREHD), Carlos III Health Institute (ISCIII), Zaragoza, Spain.; 12 Oncogenes and Tumour Metabolism Laboratory, Francis Crick Institute, London, United Kingdom.; 13 Department of Molecular Biotechnology and Health Sciences, University of Torino, Torino, Italy.; 14 Pancreatic Cancer Heterogeneity, Candiolo Cancer Institute - FPO – IRCCS, Torino, Italy.; 15 Department of Surgery, Tri-Service General Hospital, National Defense Medical Center, Taipei, Taiwan.; 16 Department of Gastroenterology, Hospital Universitario Lozano Blesa, Universidad de Zaragoza, Zaragoza, Spain.

## Abstract

The pronounced desmoplastic response in pancreatic ductal adenocarcinoma (PDAC) contributes to the development of a microenvironment depleted of oxygen and nutrients. To survive in this hostile environment, PDAC cells use various adaptive mechanisms that may represent therapeutic targets. In this study, we showed that nutrient starvation and microenvironmental signals commonly present in PDAC tumors activate PPARδ to rewire cellular metabolism and promote invasive and metastatic properties both *in vitro* and *in vivo*. Mild mitochondrial inhibition induced by low-dose etomoxir or signals from tumor-associated macrophages altered the lipidome and triggered the downstream transcriptional program of PPARδ. Specifically, PPARδ reduced mitochondrial oxygen consumption and boosted the glycolytic capacity by altering the ratio of *MYC* and *PGC1A* expression, two key regulators of pancreatic cancer metabolism. Notably, genetic or pharmacologic inhibition of PPARδ prevented this metabolic rewiring and suppressed both invasiveness *in vitro* and metastasis *in vivo*. These findings establish PPARδ as a central driver of metabolic reprogramming in response to starvation and tumor microenvironmental cues that promotes a prometastatic phenotype in PDAC, suggesting that PPARδ inhibition could serve as a therapeutic strategy to combat PDAC progression.

**Significance:**

Nutrient starvation and microenvironmental signals activate PPARδ in pancreatic cancer to support survival and metastasis by promoting metabolic plasticity and invasiveness, providing a strong rationale for developing PPARδ-targeted therapies for pancreatic cancer.

## Graphical Abstract

**Figure:**
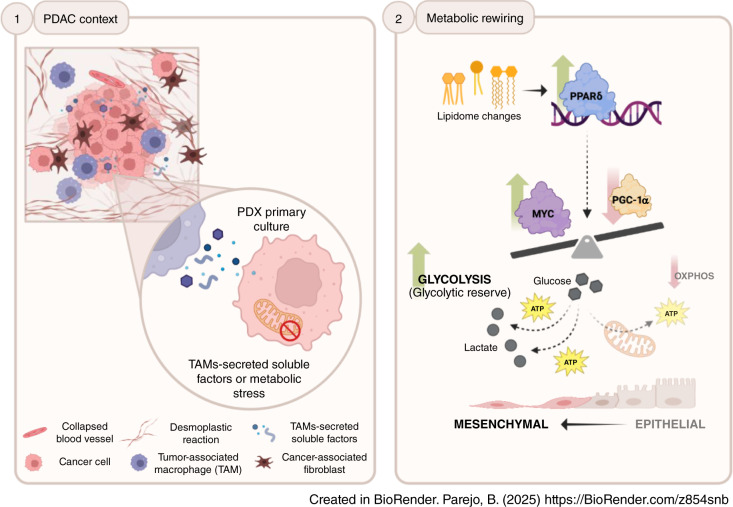

## Introduction

Pancreatic ductal adenocarcinoma (PDAC), the most frequent form of pancreatic cancer, is an extremely lethal disease with high metastatic potential (1). At the time of diagnosis, 80% to 90% of the patients have already progressed to an advanced disease stage, with very limited therapeutic options and a particularly poor long-term outcome (2). This can, at least in part, be attributed to the hierarchical organization of PDAC, containing cells with tumor-initiating properties or cancer stem cells (CSC), which constitute the driving force for disease progression, metastasis, and chemoresistance (3, 4).

We have reported that *c-MYC* (hereinafter referred to as *MYC*) plays an essential role in defining the metabolic phenotype and stemness of PDAC cells by negatively controlling the expression of the mitochondrial biogenesis factor *PPARGC1A* (peroxisome proliferator–activated receptor γ coactivator 1-α, hereinafter referred to as *PGC1A*; ref. 5). Reduced *MYC* expression in CSCs is required to unleash *PGC1A* expression to promote oxidative phosphorylation (OXPHOS), a metabolic phenotype necessary to promote their self-renewal capacity. This renders CSCs particularly sensitive to mitochondrial targeting (i.e., metformin), whereas differentiated cancer cells, characterized by increased *MYC* expression and a glycolytic phenotype, were mostly resistant to metformin (5).

Notably, however, a subset of CSCs with reduced mitochondrial content proved to be resistant to mitochondrial targeting by metformin due to an increased *MYC/PGC1A* ratio allowing them to divert glucose metabolism to glycolysis. This subset of metformin-resistant CSCs displayed a highly invasive phenotype, suggesting a potential link between the observed metabolic switch and enhanced invasiveness in response to energy deprivation and metabolic stress.

In this study, we now conclusively demonstrate that PPARδ (peroxisome proliferator–activated receptor δ) activation precedes and facilitates the acquisition of a prometastatic state in PDAC cancer (stem) cells, characterized by metabolic plasticity and epithelial-to-mesenchymal (EMT)-like features. This phenotype could be induced by both direct nutrient starvation prompted by partial mitochondrial inhibition or tumor microenvironmental cues. Intriguingly, single-cell RNA sequencing (scRNA-seq) identified PPARδ as a directly druggable upstream target, which integrates both starvation and tumor microenvironmental signals to modulate cellular metabolism and invasiveness via increasing the *MYC/PGC1A* ratio. Therefore, pharmacologic targeting of PPARδ represents a novel and translatable approach to counteract PDAC progression and metastasis.

## Materials and Methods

### Primary human PDAC cells

Tissue fragments from low-passage patient-derived xenografts (PDX) were minced and digested with collagenase [Stem Cell Technologies; 90 minutes at 37°C (6)], and after centrifugation (5 minutes, 1,200 rpm), the pellets were cultured in RPMI (Gibco, Life Technologies), 10% FBS, and 50 U/mL penicillin/streptomycin. Circulating tumor cells from patients with advanced PDAC were isolated from peripheral blood and expanded as primary cultures as previously described (7). For more information on primary cells, see Supplementary Table S1. Primary cultures were used between passages 5 and 15. For experiments, cells were cultured in DMEM:F12 supplemented with B-27, L-glutamine (all from Gibco), penicillin/streptomycin, and bFGF (PeproTech). All established primary cultures were authenticated by short tandem repeat analysis and tested for *Mycoplasma* contamination periodically.

### CSC-enriching culture

PDAC spheres were generated by seeding 10^4^ cells/mL in ultralow-attachment plates (Corning) as described previously (8).

### Primary human macrophages and conditioned media

Leukocyte cones from anonymous healthy donors were obtained from the National Blood Transfusion Service (United Kingdom) according to City and East London Research Ethics Committee (17/EE/0182). Cones were stored at 4°C and used within 24 hours of delivery. Monocyte culture, polarization into M2-like macrophages, and generation of conditioned medium were as previously described (9, 10).

### Coculture of PDAC and macrophages or cancer-associated fibroblasts

The 10^5^ M2-like polarized macrophages or primary cancer-associated fibroblasts were seeded to a presoaked 6-well 0.4-μm permeable polycarbonate membrane transwell (Corning) in Iscove’s Modified Dulbecco’s Medium (Gibco) supplemented with 10% Human Serum (Sigma-Aldrich). In parallel, 1.5 × 10^5^ PDAC cells were seeded in 6-well plates (adherent) or ultralow-attachment plates (spheres) in supplemented DMEM:F12. Cocultures were maintained in supplemented DMEM:F12 for 4 days.

### Chemicals

See Supplementary Table S2.

### Lentiviral constructs

Lentiviruses with the silencing/overexpression plasmids shown in Supplementary Table S3 were generated as previously described (5).

### Promoter reporter assays

HEK293T cells were transfected with 1 μg of the corresponding reporter plasmids (Supplementary Table S3) and Lipofectamine 2000 according to the manufacturers’ protocol. After 8 hours, PPARδ was induced by treatment with 5 μmol/L GW0742 or overexpression with 2 ng/mL doxycycline, and promoter activity was read as previously described (5).

### Lactate production

After treatments, cell culture supernatants were treated following the manufacturer’s instructions (Lactate Assay Kit II, Sigma-Aldrich).

### Flow cytometry and cell sorting

CSCs identified with an anti-CD133/1-PE (Miltenyi Biotec, cat# 130-113-108, RRID:AB_2725937) were further subdivided depending on their mitochondrial content after staining (10 minutes, 37°C) with 100 nmol/L Mitotracker Deep Red (Life Technologies). For glucose uptake, cells were incubated with 100 mmol/L 2-(N-(7-nitrobenz-2-oxa-1,3-diazol-4-yl)amino)-2-deoxyglucose (Life Technologies; 20 minutes, 37°C). DAPI (4',6-diamidino-2-phenylindole) was used for exclusion of dead cells. Cells were analyzed using a LSR Fortessa Cell Analyzer platform (BD Biosciences) or sorted using the BD FACSAria Fusion Cell Sorter (BD Biosciences, RRID: SCR_025715). Data were analyzed with FlowJo (Tree Star Inc., RRID: SCR_008520).

### XF extracellular flux analysis

Single-cell suspensions from secondary spheres/adherent cultures were plated in XF96 cell culture microplates previously coated with Cell-Tak (BD Biosciences) at 30,000 cells/well. The assays were performed following the manufacturer’s instructions, as previously described (5). Unless indicated, all reagents and materials were from Agilent Technologies.

### Invasion assay

Invasion assays were performed using 24-well 8.0 μm PET membrane invasion chambers coated with growth factor reduced Matrigel (Corning) as previously described (11). After 48 hours of pretreatment, 10^5^ primary PDAC cells were seeded to inserts in serum-free media. Invasion toward 20% FBS was tested after 12 to 24 hours of incubation at 37°C. Stained invaded cells were imaged on the Olympus BX51 Fluorescence Microscope (RRID: SCR_018949) and analyzed using ImageJ.

### PPAR activity assay

PPAR-specific DNA-binding activity was performed on nuclear extracts upon 24 hours of treatment using PPAR Transcription Factor Assay Kit (Cayman) following the manufacturer’s instructions.

### Single-cell capture, library preparation, and RNA-seq

The samples were labeled with cell hashing antibodies following manufacturer’s instruction (BioLegend), and up to 25,000 cells were loaded per lane on 10X Chromium microfluidic chips (10X Genomics). Single-cell capture, barcoding, and library preparation were performed using the 10X Chromium Single Cell 3′ Reagent Kits version 3 chemistry according to the manufacturer’s protocol (#CG000185). cDNA and HTO libraries were checked for quality on the Agilent 4200 TapeStation and quantified by KAPA qPCR before sequencing on a single lane of a NovaSeq 6000 S4 flow cell (Illumina) to an average depth of 100,000 reads per cell.

### Single-cell data processing, quality control, and analysis

The Cell Ranger pipeline (version 1.3, 10X Genomics) was used to first convert Illumina base call files to FASTQ files, and then demultiplexing was conducted before aligning FASTQs to the GRCh38 genome reference and producing the digital gene–cell count matrix. Samples were combined using the Cell Ranger aggregate function. Potential doublets were identified by DoubletFinder (12) and removed. Quality control, normalization, clustering, dimensionality reduction, and visualization were performed using R toolkit Seurat package (13). Gene–cell matrices were filtered to remove cells with fewer than 500 unique molecular identifier counts and 500 detected genes or with more than 15% mitochondrial gene counts. Gene set enrichment analysis (GSEA) was conducted using ssgsea function from the gene set variation analysis (GSVA) package.

### Bulk RNA-seq analysis

The paired-end RNA-seq libraries were generated using TruSeq Stranded mRNA kits with 200 ng of total RNA per sample. After quality control, reads were aligned to the human genome build 38 and GENCODE gene annotation human release 27 using STAR (version 2.5.3a; ref. 14). The number of reads that map to each gene was quantified by HTSeq (version 0.9.1, RRID: SCR_005514). Differential expression analysis was carried out using R/Bioconductor package DESeq (version 3.4.4, RRID: SCR_000154). The RNA-seq data were deposited at Gene Expression Omnibus (GSE135686). Differentially expressed genes identified in the macrophage-conditioned medium and etomoxir treatments were subjected to unsupervised hierarchical clustering using Pearson correlation distance matrix and complete linkage. Gene set enrichment was performed using GSEA (RRID: SCR_003199) from the Broad Institute using the Hallmark gene set database.

### RNA preparation and RT-qPCR

Total RNA and reverse transcription were performed as previously described (15). For primers information, see Supplementary Table S4. When indicated, a PCR array for 188 carbohydrate metabolism–related genes was used (5).

### Cleavage under targets and tagmentation

Cleavage under targets and tagmentation (CUT&Tag) was performed on PDAC-020 cells treated with vehicle or etomoxir for 24 hours. The procedure was performed as described previously (16). For antibody information, see Supplementary Table S5.

### *In vivo* metastasis and treatments

For classic metastasis assay upon intrasplenic injection, pretreated PDAC-354 CMV-Luciferase-RFP-TK–expressing cells were resuspended in 30 μL of Matrigel and injected in the spleen of 6-week-old NOD.CB17-Prkdcscid/NcrCrl mice (Charles River Laboratories, RRID: IMSR_CRL:394) at a concentration of 0.5 × 10^5^ cells per injection (*n* = 32 female mice, weights 18–20 g, attrition rate 12.5%). After 7 days, splenectomy was performed. For spontaneous metastasis assay, 10^5^ PDAC-265 cells were resuspended in 30 μL of Matrigel and injected orthotopically to 6-week-old NOD.CB17-Prkdcscid/NcrCrl mice (*n* = 20 female, *n* = 20 male; weights 20–25 and 25–30 g, respectively; attrition rate 5%). Mice were imaged weekly using the Perkin Elmer IVIS (RRID: SCR_018521). Mice were randomized before treatments. When indicated, mice were treated for three consecutive days with GW0742 (0.3 mg/kg i.v.) after surgery. When shPPARD cells were injected, mice were treated with oral doxycycline (2 mg/mL drinking water, Acros Organics) and etomoxir (15 mg/kg, i.p. daily, Sigma-Aldrich) for 7 days after intrasplenic implantation. When indicated, mice were treated daily with vehicle (PBS), the PPARδ agonist GW0742 (0.3 mg/kg i.p., Cayman Chemical), or the PPARδ antagonist GSK3787 (3 mg/kg i.p., Cayman Chemical) until sacrifice. Once a minimum of 10^6^ region of interest bioluminescence in liver was achieved in at least 3 mice or signs of ascites developed, all mice were sacrificed (9 weeks). Livers and pancreas were harvested and fixed in 4% paraformaldehyde. Procedures were conducted following ARRIVE guidelines and in accordance with national and Institutional Animal Care and Use Committee guidelines [Animals in Science Regulation Unit, Home Office Science, London, United Kingdom; Project License PPL70/8129; Ethical Conduct in the Care and Use of Animals as stated in The International Guiding Principles for Biomedical Research involving Animals (Council for International Organizations of Medical Sciences (CIOMS); Universidad de Zaragoza Ethics Committee; project licenses PI22/17 and PI41/20].

### Human PDAC tissue microarray

All human tissue samples and associated clinical parameters were provided by the tissue bank of the University Medical Center Mainz in accordance with the regulations of the tissue biobank and the approval of the ethics committee of the University Medical Center Mainz (approval no. 2019-14390; State of Rhineland-Palatinate Medical Chamber). Written informed consent was obtained from all patients, and the study was performed in accordance with the ethical guidelines included in the declaration of Helsinki. For further evaluation, a tissue microarray was created from *n* = 108 tumor cases, which comprises four tumor cores with a diameter of 1 mm with tissue from the center and periphery of the PDAC tumor, if available.

### IHC

Formalin-fixed, paraffin-embedded tissues were cut into 3-μm-thick serial sections, followed by deparaffinization and tissue rehydration.

For single staining, PPARδ or CK-19 antibodies (Supplementary Table S5) were diluted in Dako EnVision FLEX Antibody Diluent (Dako; Agilent Technologies) at 1:200 or 1:1,000, respectively, and incubated for 1.5 hours at room temperature. To overcome unspecific peroxidase reaction, DakoEnVision Flex Peroxidase Blocking Reagent was applied. The secondary antibodies were incubated for 0.5 hours at room temperature, followed by a color reaction with Dako EnVision Flex Substrate Buffer and Dako EnVision Flex DAB+ Chromogen according to the manufacturer’s protocol.

For double stainings, slides were incubated at room temperature with a HIF1α antibody at 1:1,000 for 0.5 hours or a vimentin antibody at 1:200 for 1 hour, followed by PPARδ or MYC at 1:200 overnight at +4°C. For visualization of antibody binding, we used Dako EnVision Flex DAB+ Chromogen and Dako EnVision Flex+ Mouse according to the manufacturer’s protocol. The slides were digitized using a Hamamatsu NanoZoomer S60 Digital slide scanner (RRID: SCR_022537). Evaluation was made with the software based using NDP.view2 (RRID: SCR_025177) and QuPath (RRID: SCR_018257) software. IHC was quantified using the widely used Allred immunoreactive score (17).

### Lipidomic analyses

All solvents were high-performance liquid chromatography–grade or LC-MS–grade purchased from VWR International GmbH and Merck KGaA. Lipids from the cells were extracted by the addition of 500 μL extraction mix (CHCl_3_/MeOH 1/5 containing appropriate internal standards). Subsequent extraction and analyses were as previously described (18). Lipid subtypes were data-mined and classified using the *lipidr* package in R Project for Statistical Computing (R, RRID: SCR_001905). Plots generated from this analysis were represented using the *ggplot2* (RRID: SCR_014601) package.

### GC-MS metabolomics

Cells were equilibrated in unlabeled tracing medium (DMEM without pyruvate, with 10 mmol/L glucose, 2 mmol/L L-glutamine, penicillin/streptomycin, bFGF, and B-27) for 3 hours, and then the medium was substituted with 4 mL/sample of tracing medium with 10 mmol/L U-^13^C6-glucose (Cambridge Isotope Laboratories). Medium samples were collected for analysis at the indicated time points. After 24 hours, cells were washed with cold PBS and scrapped in dry ice-cold MeOH. Metabolite extraction and GC-MS were performed as previously described (5).

### Western blot

Cell lysates were quantified and analyzed by Western blot as previously described [15). For antibody information, see Supplementary Table S5.

### Statistical analysis

The results for continuous variables are presented as means ± SEM unless stated otherwise. Treatment groups were compared using the unpaired two-tailed *t* test. Pair-wise multiple comparisons were performed using the two-sided one-way ANOVA with Bonferroni adjustment. *P* values < 0.05 were considered statistically significant. All analyses were performed using GraphPad Prism 8 (RRID: SCR_002798).

### Data availability

Expression data from human PDAC and normal tissues were analyzed using the webserver Gene Expression Profiling Interactive Analysis 2 [The Cancer Genome Atlas (TCGA) and the GTEx project databases; http://gepia2.cancer-pku.cn/, RRID: SCR_026154] as previously described (15). The samples included in the top and bottom quartiles of *PPARD* expression in the TCGA dataset were compared with GSEA with 1,000 permutations and FDR < 25%. Bulk RNA-seq data are available in Gene Expression Omnibus at GSE135686. scRNA-seq data are available at the NCBI dbGaP under accession numbers GSE184871, GSM5599107, and GSM5599108. All other data are available upon request from the corresponding author.

## Results

### Metabolic starvation of PDAC cells induces an EMT-like phenotype and diminishes mitochondrial activity

We have previously showed that prolonged treatment of PDAC cultures with the mitochondrial complex I inhibitor metformin eliminated the majority of CSCs but still allowed the outgrowth of preexisting resistant CSC clones (5). Metformin-resistant cells were morphologically distinct with an elongated shape and diminished cell-to-cell contact and showed upregulation of the EMT genes *VIM* and *ZEB1* (Supplementary Fig. S1A). In line with previous studies (9–11, 19), induction of these two genes accompanied with morphologic changes in response to microenvironmental signals from M2-like tumor-associated macrophages (TAM) and pancreatic stellate cells (PSC) is indicative of an EMT-like phenotype and results in invasion and metastasis in PDAC (Supplementary Fig. S1B).

**Figure 1.**
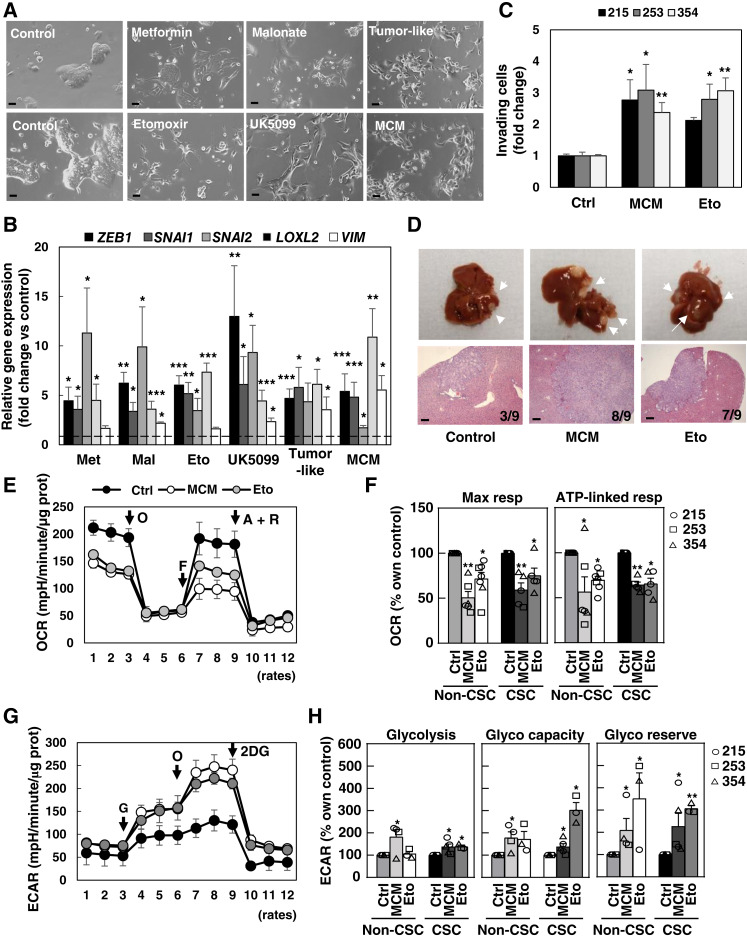
Induction of EMT-like phenotype and metabolic switch in PDAC cells upon starvation. **A,** Representative images illustrating morphologic changes for PDAC-354 cells in response to treatment for 72 hours with the complex I inhibitor metformin (3 mmol/L), the β-oxidation inhibitor etomoxir (20 μmol/L), the complex II inhibitor malonate (5 mmol/L), the pyruvate carrier inhibitor UK5099 (100 μmol/L), or tumor-like conditions [low pH (HCl 50 μmol/L) + low glucose (1 mmol/L) + 3%O_2_]. Scale bar, 50 μm. **B,** Expression of EMT-associated genes (*ZEB1*, *SNAI1*, *SNAI2*, *LOXL2*, and *VIM*) was determined by RT-qPCR after cells were treated for 48 hours as indicated in **A** or with MCM. Pooled data for PDAC-185, A6L, 215, 253, and 354 (*n* ≥ 4 for each cell type). Data are normalized to *HPRT1*. Eto, etomoxier; Mal, malonate; Met, metformin. **C,** PDAC-215, 253, and 354 cells were treated with MCM or 20 μmol/L etomoxir for 48 hours and seeded in modified Boyden invasion chambers containing 20% FBS in the lower compartment. The number of invasive cells was analyzed after 16 hours (*n* = 7–11). **D,** GFP^+^-Luciferase^+^-PDAC-354 cells were treated with control, MCM, or 20 μmol/L etomoxir for 48 hours and then injected intrasplenically to assess their metastatic capacity (*n* = 9 mice/group). Representative photographs of liver metastasis (top) and subsequent hematoxylin and eosin staining (bottom). Scale bar, 200 μm. **E,** Representative OCR profile for PDAC-253 cells treated for 48 hours as indicated (mitochondrial stress test). O, ATP synthase inhibitor oligomycin; F, mitochondrial OXPHOS uncoupler FCCP [carbonyl cyanide-4 (trifluoromethoxy) phenylhydrazone]; A+R, complex III inhibitor antimycin A + electron transport change inhibitor rotenone. **F,** Maximal and ATP-linked respiration (resp) in non-CSC vs. CSC cells. Bars represent pooled data from PDAC-215, 253, and 354, showing individual data points corresponding to each PDX (*n* = 5–7). **G,** Representative extracellular acidification rate (ECAR) profile for PDAC-253 cells treated for 48 hours as indicated (glycolysis test). G, glucose; O, ATP synthase inhibitor oligomycin; 2DG, glycolysis inhibitor 2-deoxy-glucose. **H,** Glycolysis, glycolytic capacity, and glycolytic reserve in adherent (non-CSC) vs. sphere-derived cells (CSC). Bars represent pooled data from PDAC-215, 253, and 354, showing individual data points corresponding to each PDX (*n* = 4–5). All data are represented as the mean ± SEM. *, *P* < 0.05; **, *P* < 0.01; ***, *P* < 0.001. See also Supplementary Figs. S1–S3.

It has been proposed that EMT may represent an adaptive response of cancer cells to nutrient starvation or pseudo-starvation triggers, including metabolic inhibitors such as metformin (20, 21). To determine whether the acquisition of an EMT-like phenotype could be a general downstream response to the induction of metabolic stress, we next treated various primary PDAC cultures using distinct means to either reduce mitochondrial uptake of different carbon sources or diminish the activity of the electron transport chain. Indeed, starvation conditions mimicking the tumor microenvironment (low pH, glucose and glutamine deprivation, and hypoxia) or pseudo-starvation by short-term treatment with metformin, malonate (complex II inhibitor), low-dose etomoxir [mitochondrial long-chain fatty acid (FA) transporter blocker] and UK5099 (mitochondrial pyruvate carrier blocker) resulted in morphologic and gene expression changes comparable with the ones observed upon incubation with M2-like macrophage-conditioned medium (MCM), a well-established inducer of EMT, invasion, and metastasis in PDAC (Fig. 1A and B; Supplementary Fig. S1C; refs. 9–11). Partial impairment of mitochondrial energy production by low-dose etomoxir equally induced *in vitro* invasiveness and *in vivo* metastasis (Fig. 1C and D). Of note, complete inhibition of mitochondrial FA transport using high-dose etomoxir resulted in toxicity (Supplementary Fig. S2A).

Interestingly, not only treatment with etomoxir but also with MCM reduced mitochondrial oxygen consumption rate (OCR; Fig. 1E and F). These findings indicate that the induction of an EMT-like phenotype by diverse external factors, such as starvation or tumor microenvironmental signals, is associated with reduced mitochondrial activity. Notably, these treatments consistently increased *ZEB1* expression in both CD133^+^ CSCs and CD133^–^ non-CSCs, regardless of their mitochondrial content (Supplementary Fig. S2B) and with no impact on self-renewal (Supplementary Fig. S2C). These data indicate that the induction of an EMT-like phenotype was not restricted to specific cellular subpopulations. Indeed, both maximal and ATP-linked OCRs were inhibited by 40% to 50% upon these treatments, with similar changes in sphere-derived CSC-enriched and adherent non-CSC cultures (Fig. 1E and F), despite distinct baseline respiratory rates (5). These effects on the OCR could also be induced by coculturing cancer cells with primary human TAMs or PSCs (Supplementary Fig. S3A–S3C). Conversely, metabolic parameters associated with enhanced glycolytic activity (glycolysis and glycolytic capacity and reserve) were increased in response to MCM or etomoxir in both CSCs and non-CSCs (Fig. 1G and H). Of note, these metabolic changes related to glycolysis were less evident, corroborated by a slight enhancement of glucose uptake and release of lactate and alanine upon treatments (Supplementary Fig. S3D–S3F). However, both glycolytic capacity and reserve, as indicators of metabolic plasticity, defined as the ability to switch to alternative pathways upon complete inhibition of mitochondrial ATP, were increased upon treatment with etomoxir and MCM in non-CSC and CSC-enriched cultures (Fig. 1G and H).

**Figure 2.**
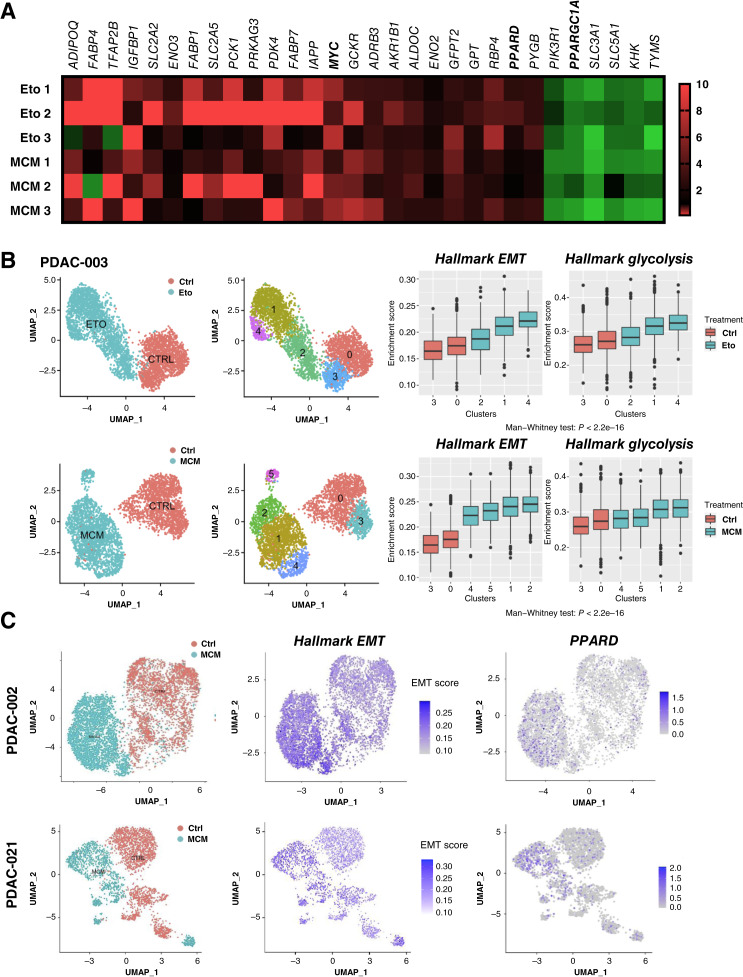
Transcriptomic and scRNA-seq analyses identify metabolic switch and EMT induction upon pseudostarvation. Cells were treated for 48 hours with vehicle (Ctrl), MCM, and 20 μmol/L etomoxir (Eto). **A,** Gene expression profile as assessed by a carbohydrate metabolism PCR array in PDAC-354 cells. Heatmap showing only genes whose expression was significantly altered (*n* = 3). **B,** Left, PDAC-003 cells were treated as indicated and were then subjected to scRNA-seq (10X Genomics Chromium platform). Unsupervised clustering of viable PDAC cells exposed to Ctrl, MCM, or etomoxir, represented as Uniform Manifold Approximation and Projection (UMAP) plots. Different clusters are color-coded. Right, boxplots illustrating gene set enrichment results for the EMT and glycolysis (Hallmark data set) for different clusters in Ctrl vs. MCM and etomoxir treatment, respectively. Differences in enrichment scores between treatments were assessed using the Mann–Whitney *U* test. **C,** Expression of EMT hallmark signature and *PPARD* in single cancer cells (PDAC-002 and 021) displayed as unsupervised clusters and color-coded for allocated treatment. See also Supplementary Figs. S4 and S5.

Next, using a carbohydrate metabolism PCR array, we identified genes implicated in the uptake and intermediary metabolism of alternative sugars such as fructose, tricarboxylic acid (TCA) substrates, amino acids, and lipids as most commonly upregulated following treatment with either MCM or etomoxir. We also observed a switch in the *MYC/PGC1A* balance toward increased *MYC* expression and decreased P*GC1A* (Fig. 2A). The increased glycolytic capacity and reserve at the expense of OXPHOS (Fig. 1E–H), together with the increased *MYC/PGC1A* ratio, seamlessly mirror our previous results in metformin-resistant primary PDAC cells (5).

We therefore hypothesized that induction of a metabolic switch and display of an EMT-like phenotype are closely associated events in response to different environmental cues, resulting in an invasive phenotype with increased metabolic plasticity. Although this phenotypic switch was detected in all subpopulations, this might be particularly relevant for CSC functionality as most of these cells in their native state lack metabolic plasticity and are unable to compensate mitochondrial impairment by switching to glycolysis (5).

### A common transcriptional program linked to PPARδ controls EMT induced by environmental signals

In order to detect specific transcriptional changes induced by the selected environmental triggers MCM and etomoxir, we next performed scRNA-seq in three different PDAC models. The results suggested that the majority of cells indeed responded by strong induction of the Hallmark EMT signature, whereas a smaller subset of cells did not respond to these cues (e.g., cluster 2 for etomoxir, Fig. 2B). These findings are consistent with the observed diverse morphologic changes upon treatment in which a subset of cancer cells maintains their epithelial morphology (Supplementary Fig. S4A). As expected, based on their discrete mechanism of action, distinct transcriptional profiles were noted for MCM and etomoxir (Supplementary Fig. S4B). However, gene set activity score analysis still revealed commonly and consistently activated metabolic pathways (glycolysis and hypoxia) as well as inflammatory signals (TNFα), an effect that was mostly confined to cells with induced Hallmark EMT signature (Fig. 2B; Supplementary Fig. S4C and S4D). Bulk transcriptional analysis showed a similar trend (Supplementary Fig. S5A and S5B), although differences were less pronounced, most likely due to contained cells that did not respond to the treatments and remained in an epithelial state. Together, these data demonstrate that the majority of PDAC cells undergo consistent transcriptional changes in response to pharmacologic/nutritional starvation and tumor microenvironmental cues, involving common metabolic changes and EMT induction.

Upon further analysis of the scRNA-seq data sets to identify specific metabolism-related genes and potential upstream regulators, we noted a consistent upregulation of the nuclear peroxisome proliferator–activated receptor-δ (*PPARD*) across the different clusters in response to both etomoxir and MCM (Fig. 2C), which was also detectable in the PCR array data (Fig. 2A). Whereas *PPARD* upregulation was heterogeneous, its expression was mostly confined to cells displaying the Hallmark EMT signature (Fig. 2C; Supplementary Fig. S6A), suggesting a critical role for *PPARD* in the EMT process. PPARδ is a member of the PPAR subfamily of nuclear hormone receptors, together with PPARα and PPARγ. This subfamily modulates energy homeostasis by controlling the expression of numerous genes involved in lipid and glucose metabolism (22). Notably, we only found *PPARD* to be consistently upregulated in EMT cells, whereas the expression of *PPARA* and *PPARG* was not altered (Supplementary Fig. S6B).

**Figure 3.**
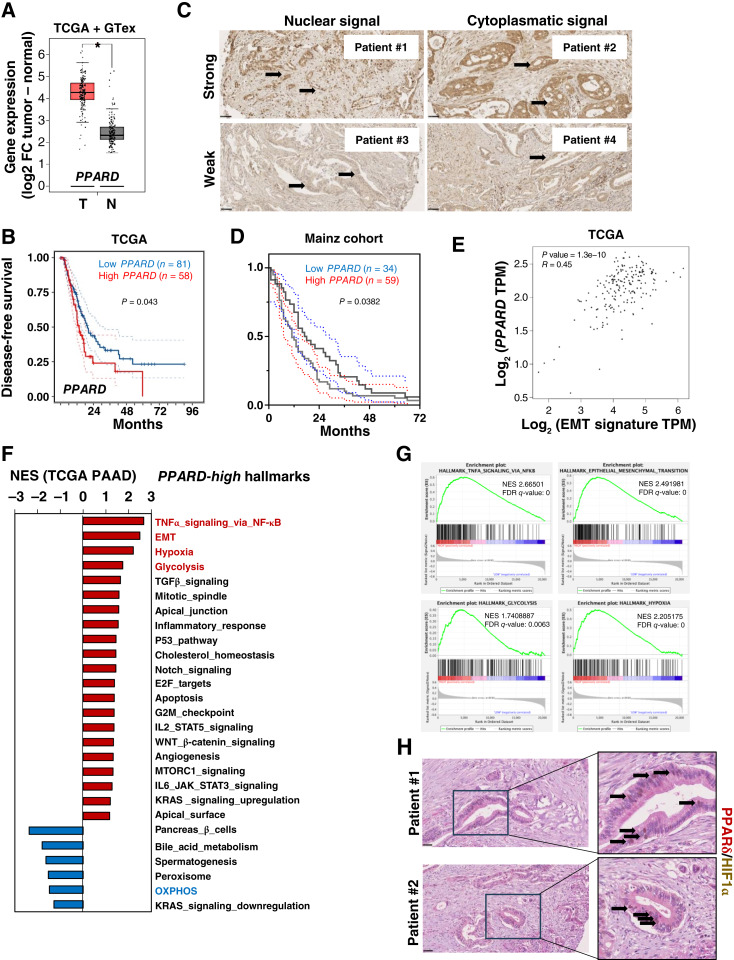
*PPARD* expression is linked to metabolic switch and EMT in patients with PDAC. **A,** Expression levels of *PPARD* in PDAC tumors (T) vs. surrounding normal tissue (N) included in the TCGA and GTEx projects. **B,** Patients were dichotomized for *PPARD* expression [higher (*n* = 58) and lower (*n* = 81) expression compared with the mean; RNA-seq V2 RSEM values]. Kaplan–Meier curves for disease-free survival are shown. Dotted lines denote the confidence intervals. Survival, 19.48 vs. 13.53 months. **C** and **D,** A TMA with 108 cases was stained by IHQ using an anti–PPARδ antibody. Representative images depicting different signal intensities (weak vs. strong) and localizations (nuclear vs. cytoplasmatic) are shown in **C**. Magnification, ×400. Scale bar, 50 μm. Arrows, stained cancer cells. **D,** Signal intensity was scored by Allred immunoreactive score, and patients were dichotomized into low (*n* = 34; scores 0–6) vs. high (*n* = 59; scores 7–8) PPARδ expression groups. Kaplan–Meier curves for overall survival are shown. Dotted lines denote the confidence intervals. Survival, 17.5 vs. 11 months. **E,** Correlation between *PPARD* tumor expression levels family members and an EMT-associated signature composed of *SNAI1*, *SNAI2*, and *ZEB1*. **F,** Gene sets enriched in the transcriptional profile of tumors belonging to the top *PPARD* high-expression group compared with the bottom low-expression group in the TCGA data series (PAAD, pancreatic adenocarcinoma). Shown are the normalized enrichment score (NES) values for each pathway using the Hallmark gene sets, meeting the significance criteria: nominal *P* value of < 0.05, FDR < 25%. **G,** Enrichment plot for TNFα, EMT, glycolysis, and hypoxia hallmarks in *PPARD*-high vs. -low samples shown in **F**, indicating values of normalized enrichment score and FDR *q* values. **H,** Representative images of costaining of PPARδ (nuclear and cytoplasmatic, red) and HIF1α (nuclear brown) by IHQ in several patients shown in **C** and **D**. Magnification, ×400. Scale bar, 50 μm. Arrows, costained cancer cells. See also Supplementary Fig. S6.

We next performed a series of bioinformatic analyses of publicly accessible human datasets to further interrogate a possible association of these nuclear receptors with human PDAC aggressiveness and metastasis. Analysis of the TCGA and GTEx datasets (http://gepia.cancer-pku.cn/index.html) showed significantly increased expression levels for *PPARD* and *PPARG* in tumoral versus normal tissue (Fig. 3A; Supplementary Fig. S6C), which also correlated with poor outcome (Fig. 3B; Supplementary Fig. S6D). The analysis of a TMA with 108 PDAC cases showed heterogenous expression of PPARδ across patients at the protein level (Fig. 3C) and further confirmed the correlation of PPARδ expression with patient survival (Fig. 3D). Interestingly, only *PPARD* expression positively correlated with an EMT-related gene signature formed by *ZEB1*, *SNAI1*, and *SNAI2* in the tumor (Fig. 3E; Supplementary Fig. S6E). We performed GSEA of the TCGA dataset and compared the top versus the bottom *PPARD* quartiles. Applying the Hallmark gene set collection, we found that the EMT pathway was one of the most significantly enriched pathways in patients with high *PPARD* expression, together with the TNFα pathways and the metabolic pathways for glycolysis and hypoxia (Fig. 3F and G). Consistently, the OXPHOS pathway was significantly downregulated in highest *PPARD* quartile (Fig. 3F). Importantly, we found costaining of PPARδ and the hypoxia marker HIF1α in specific groups of cancer cells heavily surrounded by stromal cells, further reinforcing a direct link between both pathways (Fig. 3H). These patient data align closely with the phenotype (Fig. 1) and transcriptional expression pattern (Fig. 2) observed in our *in vitro* induced conditions. This further supports the idea that PPARδ may serve as a key regulator of a metastatic program in human PDAC, linking cellular metabolism with EMT and invasiveness in response to various environmental signals.

### PPARδ directly induces functional changes associated with invasiveness and metastasis

**Figure 4.**
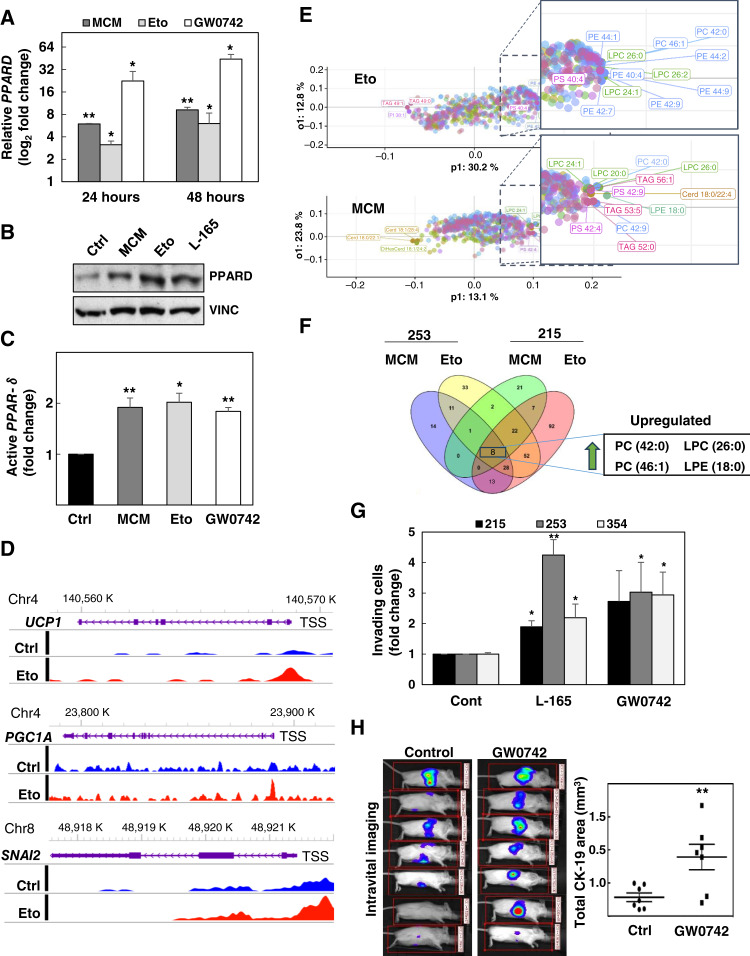
Activation of PPARδ initiates invasiveness and metastasis. **A,***PPARD* mRNA expression upon 24 to 48 hours of treatment with MCM, etomoxir (Eto), and 5 μmol/L of the PPARδ agonist GW0742. Pooled data of PDAC-215, 253, and 354 cells (*n* = 4–7). **B,** Representative Western blot after 48 hours of treatment in PDAC-354 cells. **C,** PPARδ activity, measured as binding to the PPAR response element, following stimulation with MCM, etomoxir, and the PPARδ agonist GW0742 for 24 hours (*n* = 5). **D,** CUT&Tag analysis of PPARδ protein binding at the *UCP1*, *PGC1A*, and *SNAI2* loci. WashU Epigenome browser tracks showing CUT&Tag signals at the mentioned loci with the indicated transcription start site (TSS). Blue signals represent PPARδ binding in control (Ctrl) conditions, and red signals represent PPARδ binding upon 24 hours of etomoxir treatment in PDAC-002 cells. **E,** Lipidomics analyses for PDAC-215 and PDAC-253 cells treated for 24 hours with MCM and etomoxir. OPLS-DA analysis showing the most represented lipids common for PDAC-215 and 253 for each experimental conditions vs. the control condition (*n* = 3). **F,** Venn diagram indicating the number of lipid species for each experimental group. The four common upregulated lipids for all four conditions are indicated in the square. **G,** Invasive capacity of cells treated for 48 hours with the PPARδ agonists L-165 and GW0742 (5 μmol/L). Cells were placed in modified Boyden invasion chambers containing 20% FBS in the lower compartment, and the number of invasive cells was assessed after 16 hours (*n* = 4–8). **H,** Experimental metastasis assay of PDAC-354-GFP-Luc cells pretreated with GW0742 for 48 hours. After intrasplenic injection, mice received three more daily doses of GW0742 (0.3 mg/kg i.v.). IVIS imaging (left) and quantification of the total CK-19 area in the livers 9 weeks after implantation (right). All data are represented as the mean ± SEM. *, *P* < 0.05; **, *P* < 0.01. See also Supplementary Figs. S7–S9.

Using our panel of EMT inducers shown in Fig. 1, we confirmed a consistent upregulation of *PPARD*, irrespective of the utilized trigger (Fig. 4A; Supplementary Fig. S7A). MCM and etomoxir consistently upregulated *PPARD* within 24 hours, when changes of cellular morphology were still minor or even undetectable (Fig. 4A; Supplementary Fig. S7B and S7C). These changes at the mRNA level translated into increased protein expression and activation of PPARδ as evidenced by direct binding to the PPAR response element (Fig. 4B and C), binding to regulatory sequences in the promoter of its well-known direct target *UCP1* (Fig. 4D), and upregulation of PPARδ target genes (Supplementary Fig. S7D). Interestingly, our CUT&Tag analysis also showed direct binding of PPARδ to *PGC1A* and *SNAI2* loci (Fig. 4D), both of them implicated in the prometastatic phenotype induced by starvation/pseudostarvation signals.

Interestingly, response to MCM and etomoxir was comparable with the changes induced by pharmacologic PPARδ activation using the chemical agonists GW0742 or L-165 (Fig. 4A and B; Supplementary Fig. S8A). These data suggest that canonical ligand-dependent activation of PPARδ could be the initial event of this signaling cascade. In order to identify putative natural ligands activating PPARδ, we performed lipidomic analyses on PDAC cells upon treatment with MCM and etomoxir. Whereas the changes in the lipidome caused by MCM were less pronounced than those triggered by etomoxir (Fig. 4E; Supplementary Fig. S8B), four glycerophospholipids were consistently upregulated by both treatments across two different PDAC models (Fig. 4F): phosphatidylcholines 42:0 and 46:1, lysophosphatidylcholine 26:0, and lysophosphatidylethanolamine 18:0. All four lipids have previously been related to PPARs signaling or directly linked to PPARδ activation (23–25).

**Figure 5.**
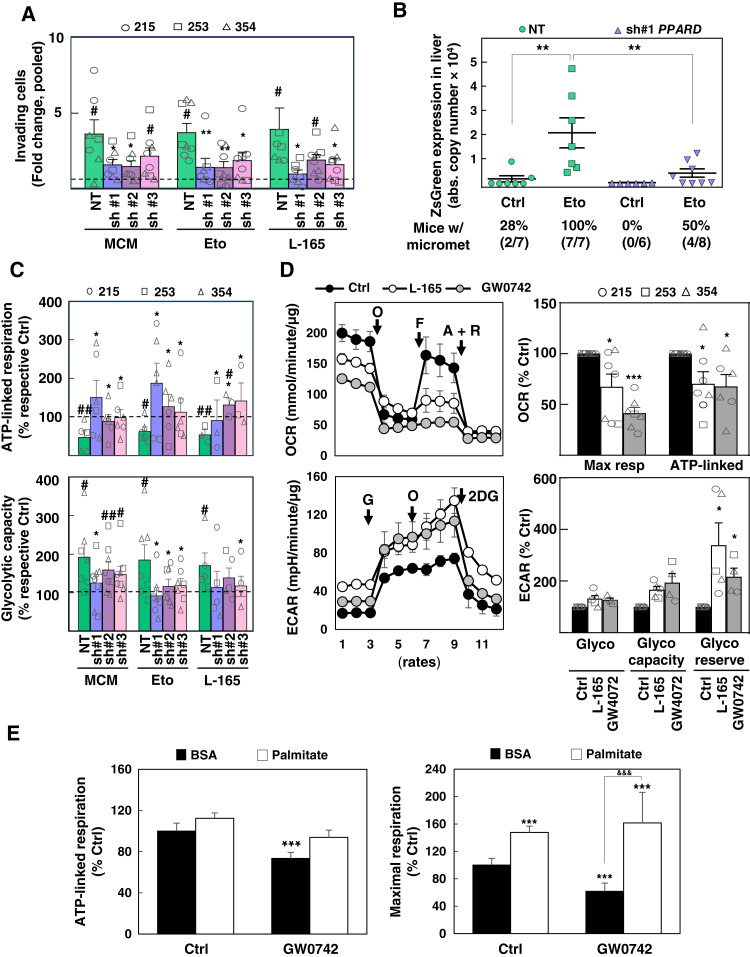
PPARδ controls the balance between OXPHOS and glycolysis, linked to EMT and metastasis. **A,***In vitro* invasion in PDAC-215, 253, and 354 cells stably transduced with inducible lentiviral vectors expressing either a nontargeting short hairpin RNA (NT shRNA) or three different shRNAs against *PPARD* (sh#1, sh#2, and sh#3). Transduced cells were pretreated with doxycycline for 24 hours, then incubated with MCM, etomoxir (Eto), or L-165 for 48 hours, and finally plated in modified Boyden chambers for 16 hours (*n* = 7). **B,** Top, ZsGreen expression by RT-qPCR in liver homogenates from an *in vivo* metastasis assay of PDAC-354 cells stably expressing either the NT or the sh#1 against *PPARD*. Cells were pretreated with doxycycline and/or 20 μmol/L etomoxir for 48 hours. After intrasplenic implantation, mice were treated with oral doxycycline (2 mg/mL; drinking water) and etomoxir (15 mg/kg, i.p. daily) for 7 days, when splenectomies were performed. Bottom, numbers indicate the percentage and total number of micrometastases in each experimental group. **C,** PDAC-215, 253, and 354 transduced cells as in **A** were pretreated with doxycycline for 24 hours, then incubated with MCM, etomoxir, or L-165, and then tested for ATP-linked respiration (top) and glycolytic capacity (bottom) after additional 24 hours (*n* = 8). **D,** Mitochondrial stress test (top row) and glycolysis test (bottom row) following treatment with control (Ctrl) or the PPARδ agonists L-165 or GW0742. Left column, representative OCR and extracellular acidification rate (ECAR) profiles for PDAC-253. Right column, pooled data for PDAC-215, 253, and 354 cells (*n* = 6–9). Glyco, glycolytic; Max res, maximum respiration; O, ATP synthase inhibitor oligomycin; F, mitochondrial OXPHOS uncoupler FCCP [carbonyl cyanide-4 (trifluoromethoxy) phenylhydrazone]; A+R, complex III inhibitor antimycin A + electron transport change inhibitor rotenone. G, glucose; 2DG, glycolysis inhibitor 2-deoxy-glucose. **E,** ATP-linked respiration (left) and maximal respiration (right) for control vs. GW0742-treated cells following treatment with or without palmitate-BSA (FAO assay). PDAC-354 cells were treated with 10 μmol/L GW0742 for 48 hours prior to the assay (*n* = 5). In **A**, **C**, and **D**, the bars represent pooled data from PDAC-215, 253, and 354, showing individual data points corresponding to each PDX. All data are represented as the mean ± SEM. *, *P* < 0.05; **, *P* < 0.01; ***, *P* < 0.001 vs. control, ###, *P* < 0.001 vs. palmitate.

Importantly, activation of PPARδ with different chemical ligands resulted in a dose-dependent induction of EMT-related genes and typical morphologic changes in diverse PDAC models (Supplementary Fig. S8A and S8C). Functionally, PPARδ activation by agonists resulted in enhanced invasiveness *in vitro* (Fig. 4G) and, most importantly, promoted metastasis *in vivo* (Fig. 4H). Conversely, knockdown of *PPARD* virtually abrogated the transcriptional changes and invasiveness induced by MCM, etomoxir, and the PPARδ agonist L-165 (Fig. 5A; Supplementary Figs. S8D and S9) and inhibited etomoxir-induced metastasis *in vivo* (Fig. 5B). Together, these data demonstrate that the metabolic regulator PPARδ is responsible for transcriptional and functional changes concomitant with EMT induction upon direct activation with chemical agonists or in response to starvation and tumor microenvironmental signals.

Given the well-established role of PPARδ in regulating cellular metabolism, we carried out a series of metabolic assessments. Functionally, when *PPARD* upregulation was inhibited using inducible knockdown, the metabolic effects of etomoxir and MCM treatment—such as reduced mitochondrial respiration and increased glycolytic capacity—were nullified (Fig. 5C). Conversely, treating PDAC cells with the PPARδ agonists GW0742 and L-165 mimicked the metabolic shift induced by etomoxir and MCM (Fig. 5D). In line with PPARs’ canonical role in stimulating FA oxidation (FAO), the reduction in mitochondrial respiration was fully reversed by adding palmitate to the culture medium (Fig. 5E). This suggests that PPARδ promotes glucose diversion to glycolysis while simultaneously upregulating the FAO machinery to provide an alternative carbon source for the TCA cycle when substrates are available.

Together, our findings support that PPARδ activation triggers a comprehensive transcriptional program that modulates cellular metabolism and induces EMT in response to various starvation and tumor microenvironmental signals.

### PPARδ downstream signaling cascade initiates a metabolic switch and promotes invasiveness

Given that MYC plays a crucial role in shaping the metabolic phenotype and stemness of PDAC cells by negatively regulating the expression of the mitochondrial biogenesis factor PGC1α (5), we hypothesized that the transcriptional program initiated by PPARδ activation might be linked to the altered balance between MYC and PGC1α expression. Initially, we observed that direct overexpression of *MYC*, previously shown to strongly suppress mitochondrial respiration and promote glycolysis, induced an EMT-like phenotype (Supplementary Fig. S10A).

**Figure 6.**
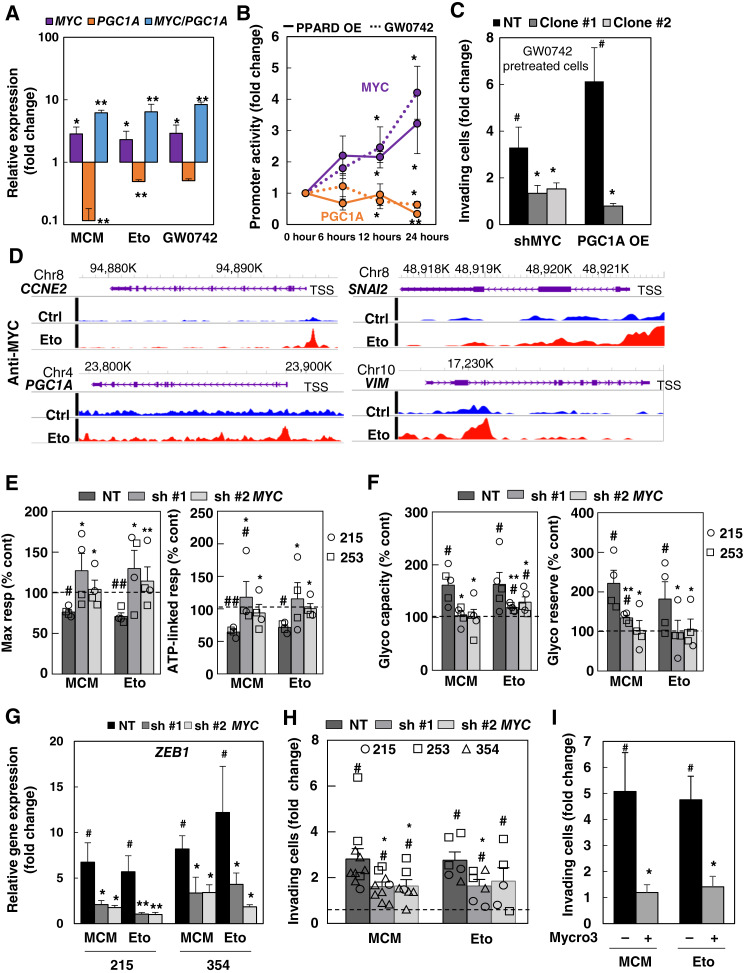
PPARδ rewires cellular metabolism regulating *MYC/PGC1A* balance. **A,** Expression of *MYC*, *PGC1A*, and *MYC/PGC1A* ratio in PDX-354 after mitochondrial energy deprivation during 48 to 72 hours (*n* = 4–7). **B,***MYC* and *PGC1A* reporter assay. Promoter activity was estimated as luciferase bioluminescence at the indicated times following treatment with PPARδ agonist GW0742 or *PPARD* overexpression (*PPARD* OE; *n* = 3–5). **C,** PDAC-354 cells were transduced with inducible lentiviral vectors expressing either a nontargeting short hairpin RNA (NT shRNA) or two different shRNAs against *MYC* (sh#1 and sh#2) or the complete cDNA of *PGC1A*. Effect of* MYC* knockdown (shMYC, pooled data for sh#1 and sh#2) or *PGC1A* overexpression (*PGC1A* OE) on invasiveness in response to treatment with 5 μmol/L PPARδ agonist L-165 for 48 hours (*n* = 6–8). **D,** CUT&Tag analysis of MYC protein binding at the *CCNE*, *PGC1A*, *SNAI2*, and *VIM* loci. WashU Epigenome Browser tracks showing CUT&TAG signals at the mentioned loci with the indicated transcription start site (TSS). Blue signals represent MYC binding in control (Ctrl) conditions, and red signals represent MYC binding upon 24 hours of etomoxir (Eto) treatment in PDAC-002 cells. **E–H,** PDAC-215 and 354 cells were transduced as in **A**, pretreated with doxycycline for 48 hours, and then incubated with MCM or etomoxir. **E,** OCR changes for maximal respiration (Max resp; left) and ATP-linked respiration (*n* = 4; right). **F,** Glycolytic (Glyco) capacity (left) and reserve (*n* = 4; right). **G,***ZEB1* gene expression. **H,** Invasive capacity (*n* = 10). **I,** PDAC-354 cells were treated with MCM or 20 μmol/L etomoxir for 48 hours in the presence or absence of the MYC/Max interaction inhibitor Mycro3 (25 μmol/L). Cells were then seeded in modified Boyden invasion chambers containing 20% FBS in the lower compartment. The number of invasive cells was assessed after 16 hours (*n* = 5). In **E**, **F**, and **H**, the bars represent pooled data from PDAC-215 and 354, showing individual data points corresponding to each PDX. All data are represented as the mean ± SEM. #, *P* < 0.05; ##, *P* < 0.01; ###, *P* < 0.001 vs. unstimulated control. *, *P* < 0.05; **, *P* < 0.01; ***, *P* < 0.001 vs. NT. See also Supplemenatry Figs. S10–S13.

We then examined *MYC* and *PGC1A* expression across various modalities of EMT induction via PPARδ activation. At the mRNA level, we found that increased *MYC* expression and a higher *MYC/PGC1A* ratio were consistently associated with EMT induction (Fig. 6A; Supplementary Fig. S10B and S10C). This finding was confirmed at the protein level, in which MCM, etomoxir, and the PPARδ agonist L-165 consistently elevated MYC expression and reduced PGC1α expression (Supplementary Fig. S10D). Notably, *PPARD* knockdown, which inhibited invasion and metastasis (Fig. 4E and F), reversed the MCM-, etomoxir-, and PPARδ agonist L-165–induced increase in the *MYC/PGC1A* ratio (Supplementary Fig. S11). Furthermore, *PPARD* overexpression or treatment with PPARδ agonists consistently enhanced *MYC* promoter activity while suppressing *PGC1A* promoter activity (Fig. 6B), establishing a direct link between PPARδ activity and the *MYC/PGC1A* expression balance. The enhanced invasiveness of cancer cells following PPARδ activation by GW0742 was reversed by *MYC* knockdown or *PGC1A* overexpression (Fig. 6C), suggesting that an elevated *MYC/PGC1A* ratio is essential for invasion. Interestingly, CUT&Tag analyses demonstrated direct MYC binding upon treatment with etomoxir not only to previously described downstream targets such as *CCNE2* and *PGC1A* but also to *SNAI2* and *VIM* (Fig. 6D), suggesting that PPARδ’s prometastatic effects are partially driven by direct transcriptional regulation of EMT-related genes by MYC.

To further validate MYC’s critical role in the metastatic program, we used an inducible *MYC* knockdown system to prevent its upregulation upon MCM and etomoxir. As anticipated, *MYC* knockdown prevented the downregulation of *PGC1A* in response treatments (Supplementary Fig. S12A) and blocked the associated metabolic shift linked to EMT induction (Fig. 6E and F) but showed little effect in basal conditions (Supplementary Fig. S12B–S12E). *MYC* knockdown also inhibited *ZEB1* upregulation and the induction of invasiveness (Fig. 6G and H; Supplementary Fig. S12D and S12E). These effects were mirrored by pharmacologic inhibition of MYC using the MYC/MAX interaction inhibitor Mycro3 (Fig. 6I; Supplementary Fig. S13A). Importantly, *PGC1A* overexpression prior to starvation prevented the metabolic changes induced by MCM and etomoxir, and as a result, the cells did not acquire an invasive phenotype (Supplementary Fig. S13B). Collectively, these data support our hypothesis that MYC, through inhibition of PGC1α, is a key mediator of the downstream effects triggered by PPARδ activation, governing both metabolic alterations and the EMT/invasive program.

### Targeting PPARδ therapeutically abolishes metastatic activity

**Figure 7.**
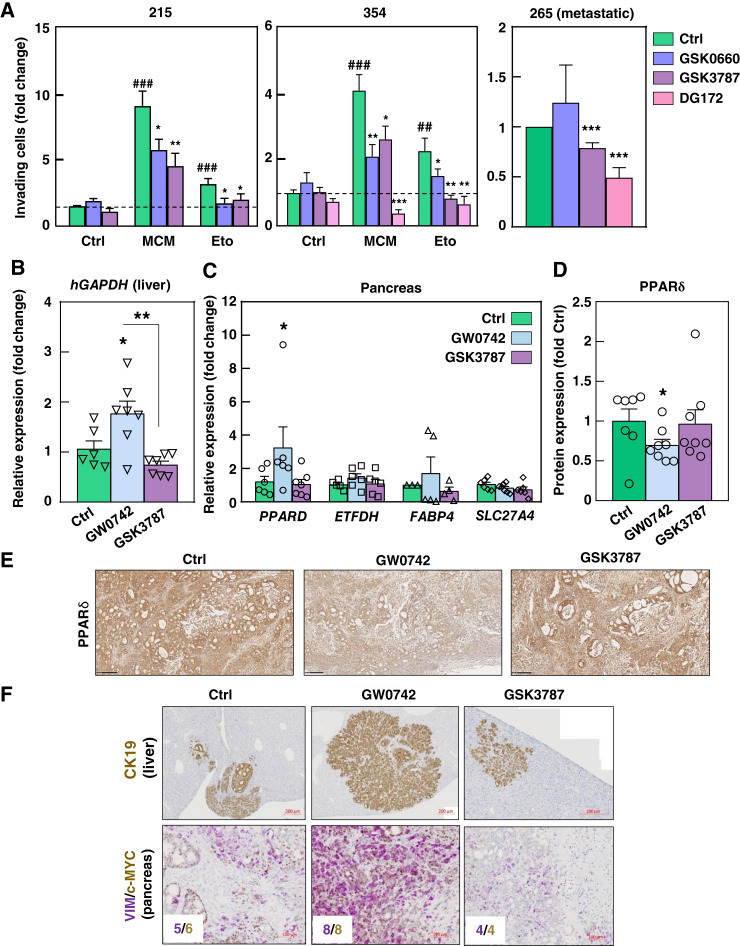
Therapeutic targeting of PPARδ impairs invasion *in vitro* and metastasis *in vivo*. **A,** PDAC-215, 354, and 265 cells were pretreated with PPARδ antagonists GSK0660 (10 μmol/L) and GSK3787 (10 μmol/L) and inverse agonist DG172 (2.5 μmol/L) for 1 hour and then treated (PDAC-215 and 354) or not (PDAC-265) with MCM or etomoxir (Eto) for 48 hours. Invasion over 16 hours was assessed in modified Boyden invasion chambers (*n* = 6). **B–F,** Spontaneous metastasis upon orthotopic injection of 10^5^ metastatic PDAC-265-GFP-Luc cells (*n* = 8 mice/group). Following implantation, mice were treated daily with either vehicle, the PPARδ agonist GW0742 (0.3 mg/kg i.p.), or the PPARδ antagonist GSK3787 (3 mg/kg i.p.) until termination of the experiment at week 9, when mice became moribund. Tumor growth was assessed by weekly IVIS. **B,** Metastasis onset evaluated as *hGAPDH* absolute copy number in livers. **C,** Expression of *PPARD* and downstream targets in pancreatic tumors measured by RT-qPCR. **D,** Quantification of PPARδ protein expression relative to β-actin that was used as loading control, measured by Western blot. **E,** Expression levels of PPARδ by IHQ (representative images). **F,** Expression levels of CK-19 in liver sections (top, representative images) or c-MYC (brown) and VIM (purple) in pancreatic tumors was measured by IHQ (bottom, representative images). MYC and VIM stainings were quantified using the Allred score, and median scores per group are shown as text inserts. All data are represented as the mean ± SEM. *, *P* < 0.05; **, *P* < 0.01; ***, *P* < 0.001 vs. control cells; #, *P* < 0.05; ##, *P* < 0.01; ###, *P* < 0.001 vs. control or single treatment. See also Supplementary Fig. S14.

Finally, we investigated whether pharmacologically blocking PPARδ could inhibit invasion and metastasis *in vitro* and *in vivo*. Pretreatment with PPARδ antagonists GSK0660 and GSK3787, or the inverse agonist DG172, effectively inhibited the invasive capacity induced by MCM or etomoxir, as well as the basal invasive capacity of highly metastatic models (Fig. 7A; Supplementary Fig. S14A). To confirm these findings *in vivo*, we used a model of spontaneous metastasis following orthotopic implantation of the metastatic PDX-derived PDAC-265 or CTCA, which rapidly formed large, unstructured tumors with extensive necrotic areas because of oxygen and nutrient deprivation. Importantly, tumors treated with the PPARδ agonist GW0742 led to increased metastatic spread, whereas treatment with PPARδ antagonists GSK3787 or GSK0660 significantly reduced metastatic dissemination (Fig. 7B; Supplementary Fig. S14B; Table 1; Supplementary Table S6). Unexpectedly, although *PPARD* mRNA was significantly elevated in tumors treated with the PPARδ agonist GW0742 (Fig. 7C; Supplementary Fig. S14C), its expression was reduced at the protein level (Fig. 7D and E). Notably, MYC and vimentin protein expression were significantly upregulated in tumors treated with GW0742 (Fig. 7F; Supplementary Fig. S14D).

**Table 1.** Pharmacologic targeting of PPARδ modulates metastasis *in vivo*.

Parameter	Control	GW0742	GSK3787
Macrometastases (%)	22	66^a^	10
Micrometastases (%)	33	87.5^a^	10^a^
Total CK area (mm^2^)	0.53 ± 0.12	1.92 ± 0.35	0.21 ± 0.11

NOTE: Table related to the data included in Fig. 7B–F. Quantification of macrometastases (percentage of livers with at least one macroscopic metastasis) and micrometastases (percentage of livers with at least one microscopic metastasis detected with CK-19 staining; total area stained for CK-19) in livers from mice treated as indicated.

a *, *P* < 0.05.

In summary, PPARδ integrates microenvironmental signals to reprogram PDAC cell metabolism via the *MYC/PGC1A* axis, thereby promoting cancer cell invasiveness and *in vivo* metastasis in PDAC. Importantly, this process can be reversed pharmacologically using existing small-molecule inhibitors, offering a promising new approach for treating advanced PDAC.

## Discussion

In most cancer types, defective vascularization and uncontrolled tumor growth lead to a lack of oxygen and nutrients within the tumor microenvironment. This issue is particularly pronounced in PDAC, in which a strong desmoplastic response exacerbates this condition, creating a persistently starved environment in which tumor cells survive through various adaptive mechanisms (26). In this study, we report that direct nutrient starvation or pseudo-starvation caused by partial inhibition of mitochondrial activity triggers an integrated response in PDAC cells, involving a metabolic switch that coincides with EMT and increased invasiveness (Fig. 1; Supplementary Fig. S1). Whereas glutamine deprivation or protein intake restriction has recently been linked to adaptive responses, including EMT, in murine PDAC cells (21, 27), we describe a broader phenomenon here: inhibition of mitochondrial uptake of various carbon substrates (such as glutamine, pyruvate, and FAs) and/or oxygen deprivation, which mimics the tumor microenvironment, consistently induced EMT. Our findings using metformin or malonate are consistent with studies in other cancer types in which electron transport chain inhibition led to EMT induction (28, 29). Interestingly, a similar phenotype can also be triggered by metabolic stress resulting from (epi)genetic inhibition of mitochondrial function, such as mitochondrial DNA depletion (30), mutations in TCA cycle enzymes (31–33), or downregulation of OXPHOS components (34), further linking starvation caused by reduced mitochondrial energy production to EMT.

Interestingly, we observed that treatment with MCM, initially included in our study as a positive control for EMT, invasion, and metastasis in PDAC cells (9–11), led to a similar reduction in mitochondrial respiration (Fig. 1E–H). As EMT induction by starvation and tumor microenvironmental signals is associated with decreased mitochondrial function, cells must rely on alternative energy sources to maintain their energy balance. Our findings show that the expected increase in glycolysis during EMT induction by mitochondrial inhibition or microenvironmental signals is relatively modest (Fig. 1G and H; Supplementary Fig. S3D–S3F). Instead, we discovered that glycolytic reserve was more significantly enhanced in EMT cells, indicating increased metabolic plasticity and diversification of metabolic substrates, such as alternative sugars or FAs (Figs. 1H and 2A). Indeed, detachment from the matrix—an early step in the metastatic process—has been previously shown to reduce mitochondrial activity and trigger metabolic adaptations like increased glycolysis or FAO (35, 36). Together, these data suggest that a metabolic switch, involving the acquisition of metabolic plasticity to compensate for diminished mitochondrial respiration, is crucial for meeting the heightened energy demands during EMT, invasion, and the subsequent metastatic process.

Our findings identify PPARδ as a key integrative sensor of various microenvironmental signals in PDAC, driving a prometastatic transcriptional program that involves both a metabolic switch and increased invasiveness. Through single-cell analysis of various PDAC primary cultures, we observed that *PPARD* was specifically upregulated in cells undergoing EMT in response to MCM or etomoxir (Fig. 2B and C). Additionally, we found that *PPARD* is overexpressed in patient datasets (Fig. 3A and C) and correlates with disease-free survival (Fig. 3B and D). Notably, *PPARD*^high^ patients also showed enrichment in pathways related to cellular metabolism, inflammation, the cell cycle, and EMT (Fig. 3E–G), consistent with our *in vitro* findings using single-cell analysis (Fig. 2; Supplementary Fig. S4). Pharmacologic and genetic approaches demonstrated that ligand-dependent activation of PPARδ leads to transcriptional changes that translate into functional alterations (Figs. 4 and 5), enabling tumor cells to (i) gain metabolic plasticity, allowing them to adapt and survive under challenging environmental conditions, and (ii) acquire mobility, facilitating their escape from the primary tumor to seek more favorable environments elsewhere.

PPARδ is part of the nuclear receptor superfamily of transcription factors and regulates various biological processes, depending on the specific cell type and context. These processes include cellular metabolism, proliferation, differentiation, survival, and inflammation (37). PPARδ can act through different transcriptional mechanisms, either repressing or activating genes in ligand-dependent and -independent manners (38). Our findings suggest that the prometastatic program driven by PPARδ in PDAC operates via ligand-dependent canonical activity, as chemical agonists like GW0742, GW501516, and L-165 replicate MCM and etomoxir effects. Although we identified potential PPARδ ligands upregulated by both stimuli in two different PDX models (Fig. 4E and F), we were unable to trigger the prometastatic pathway when these lipids were applied individually *in vitro*. Given that the overall changes in the cellular lipidome caused by MCM and etomoxir are notably different in the two independent PDXs used for these experiments (Supplementary Fig. S7E), these results suggest that a combination of ligands, not necessarily common to both treatments and, possibly, PDX-dependent, may be required to reach the activation threshold for PPARδ. Alternatively, we hypothesize that a general alteration of the lipidome could trigger this pathway in response to certain stimuli, such as specific cytokines or chemokines inducing proinflammatory lipid signaling.

The role of PPARδ in cancer remains debated (39). Whereas it has occasionally been associated with tumor suppression (40), increased *PPARD* expression has predominantly been linked to enhanced metastasis in several *in vivo* models (41). More significantly, *PPARD*^high^ patients have worse outcomes, including reduced metastasis-free survival, across various cancer types (41, 42). Contrary to some early reports (43, 44), our findings align with growing evidence that PPARδ also promotes tumor progression and metastasis in PDAC. Recent studies suggest that PPARδ activation contributes to pancreatic tumorigenesis by fostering an immunosuppressive microenvironment through the upregulation of cytokines and chemokines, which facilitates the recruitment of myeloid dendritic cells and macrophages while limiting the infiltration of CD4^+^ and CD8^+^ T cells (45, 46). In line with this, our data suggest that PPARδ’s role in the interaction between tumor cells and TAMs may be bidirectional, as we observed *PPARD* upregulation and activation in response to signals from TAMs (Figs. 2 and 4A–C). This could potentially create a positive feedback loop *in vivo*, further driving tumor progression by inducing EMT in cancer cells.

To the best of our knowledge, this is the first report linking PPAR-δ to tumor progression and metastasis through metabolic rewiring. PPARδ activates a transcriptional program that includes its canonical targets (Fig. 4D; Supplementary Fig. S7D), along with a prometastatic metabolic program by increasing the *MYC/PGC1A* ratio, which we previously identified as key regulators of the PDAC metabolic phenotype (5). Indeed, the phenotype associated with enhanced PPARδ activation was reversed by *MYC* knockdown or pharmacologic inhibition, as well as by *PGC1A* overexpression (Fig. 6; Supplementary Figs. S12 and S13). Because pharmacologic or genetic induction of *PPARD* led to a rapid upregulation of *MYC* within 24 hours and the *MYC* promoter contains a PPAR responsive element (Genecard), we initially hypothesized a direct regulation of *MYC* expression by PPARδ. However, our CUT&Tag analysis did not detect a direct binding of PPARδ in the *MYC* loci at 24 hours, when activity of the *MYC* promoter is increased (Fig. 6B). Although we cannot definitely rule out a direct regulation at earlier time points, other mechanisms such as indirect regulation via microRNA Let-7c (47) could be playing a role in our model. Nevertheless, although our results suggest that PPARδ and MYC could jointly regulate *PGC1A* and *SNAI2* expression (Figs. 4D and 6D), MYC controls additional metabolic and EMT-related genes such as *VIM* (Fig. 6D).

MYC and PGC-1α have been linked to metabolic switching and tumor progression/metastasis. Specifically, *MYC* expression promotes cellular dedifferentiation, EMT, and increased metastatic potential (48–51). In fact, the aggressive squamous/mesenchymal PDAC molecular signature depends on MYC-activated signaling pathways (52, 53). Additionally, PDAC models have shown that *MYC* overexpression is associated with less differentiated tumors and a glycolysis-related gene signature (50, 51). Conversely, reduced PGC1α expression has been shown to play a critical role in promoting migration and metastasis in melanoma and prostate cancer (54–56). Our findings demonstrate that MYC directly induces EMT-related genes such as *SNAI2* and *VIM* and suppresses *PGC1A*, leading to increased invasiveness and altered metabolism with increased global glycolytic/plastic and decreased mitochondrial oxygen consumption and activity, both likely driven by the combined regulatory effect of PPARδ and MYC.

Although both CSCs and non-CSCs can undergo EMT regardless of their basal metabolic phenotype (Supplementary Fig. S2B), CSCs are the most capable and aggressive in establishing new metastases because of their inherent self-renewal and tumor-initiating abilities (3, 4). Previously, we reported that most CSCs in the primary tumor rely on OXPHOS activity and have the highest tumorigenic potential. In this study, we extend these findings by demonstrating that CSCs undergoing EMT retain their self-renewal capacity (Supplementary Fig. S2C). Although this was an unexpected result, it suggests a complex interaction between stemness, EMT, and cellular metabolism (57). Given the importance of maintaining stemness in cancer (58, 59), we hypothesize that during EMT, PPARδ emerges as a crucial driver of stemness, making CSCs less dependent on mitochondrial metabolism. Future research should explore this potential mechanistic duality in CSCs.

Finally, we observed that genetic or pharmacologic targeting of PPARδ inhibited tumor aggressiveness and metastasis both *in vitro* and *in vivo* (Figs. 5 and 7; Supplementary Figs. S9 and S14). These findings align with previous reports from murine PDAC models, in which *Ppard* knockdown significantly reduced tumorigenesis in mouse melanoma cells (41) and suppressed tumor progression in KC mice on a high-fat diet (45). Collectively, the increasing evidence strongly supports the idea that PPARδ inhibition lowers the *MYC/PGC1A* ratio, thus curbing PDAC progression and metastasis. These data provide a strong rationale for developing novel PPARδ-targeted therapies to combat advanced pancreatic cancer.

## Supplementary Material

Table S1 Primary PDAC cells used in this study

Table S2 Main chemicals used for in vitro and in vivo treatments

Table S3 Plasmids used in this study

Table S4 Primers for rt-qPCR used in this study

Table S5 Antibodies used in this study

Table S6 Pharmacological targeting of PPAR-δ modulates metastasis in vivo

Figure S1 Different stimuli promoting mitochondrial energy deprivation or coculture with stromal cells the expression of epithelial-to-mesenchymal transition genes in PDAC cells

Figure S2 Incubation with a non-toxic dose of etomoxir and macrophage-conditioned medium does not affect CD133 expression or self-renewal of PDAC cells

Figure S3 Etomoxir or stromal signals alter different metabolic parameters in PDAC cells

Figure S4 Incubation with etomoxir and MCM induce epithelial-to-mesenchymal transition at the single-cell level

Figure S5 EMT induction by etomoxir and MCM in bulk transcriptomics

Figure S6 Different expression analyses performed for *PPARA* and *PPARG* in the TCGA database

Figure S7 Expression of the PPAR family members and downstream targets after incubation with etomoxir and MCM

Figure S8 EMT induction by PPAR-δ agonists, lipidomic changes and inhibition of EMT by downregulation of *PPARD*

Figure S9 Effects of *PPARD* knockdown in the expression of genes related with the EMT program in different PDAC PDXs

Figure S10 The EMT program is related to *MYC* upregulation and *PGC1A* downregulation

Figure S11 *PPARD* knockdown modifies the expression ratio between *MYC* and *PGC1A*

Figure S12 *MYC* downregulation reverses the prometastatic phenotype induced by etomoxir and MCM

Figure S13 Effects of MYC inhibition with mycro3 on EMT genes expression and changes induced by *PGC1A* overexpression at the functional level

Figure S14 Effects of pharmacological modulation of PPAR-δ on invasion and metastasis in vivo, using the highly metastatic PDX CTCA
