# Supplementary material for: PPARδ Orchestrates a Prometastatic Metabolic Response to Microenvironmental Cues in Pancreatic Cancer
Source: Cancer Res. 2025 Jul 3;85(17):3275–91. doi: 10.1158/0008-5472.CAN-24-3475 (PMC12402788; doi:10.1158/0008-5472.CAN-24-3475)
Supplement: Table S1 — Primary PDAC cells used in this study [file can-24-3475_table_s1_suppst1.docx]

| **Primary PDAC cells** | **Type** | **Centre of Origin** | **Country** | **MTA** |
| --- | --- | --- | --- | --- |
| PDAC-A6L, 185, 215, 253, 354, 265 | Primary PDX | Spanish National Cancer Research Centre Biobank | Spain | M-20/002-1 |
|  |  |  |  | I409181220BSMH |
|  |  |  |  | 1204090835CHMH |
| PDAC-10953 | Primary PDX | ARC-NET Biobank, 'Rossi’ University of Verona Hospital | Italy | 6.B.04 |
| PDAC-CTCA | Circulating Tumor Cell | Pancreas Tissue Bank, Barts Cancer Institute | United Kingdom | 2019/02/IISA/PS/E/Cellcultures |
| PDAC-002, 003, 006, 021, 023 | Circulating Tumor Cell | Shanghai Jiaotong University School of Medicine | China | n.a. (internal ethics protocol 20130905) |

**Table S1.** Primary PDAC cells used in this study. Information about name, type, centre of origin, country and material transfer agreement (MTA) is listed.
