# Supplementary material for: PPARδ Orchestrates a Prometastatic Metabolic Response to Microenvironmental Cues in Pancreatic Cancer
Source: Cancer Res. 2025 Jul 3;85(17):3275–91. doi: 10.1158/0008-5472.CAN-24-3475 (PMC12402788; doi:10.1158/0008-5472.CAN-24-3475)
Supplement: Table S2 — Main chemicals used for in vitro and in vivo treatments [file can-24-3475_table_s2_suppst2.docx]

| **Chemicals** | **Company** | **Solvent** | **Concentration** |
| --- | --- | --- | --- |
| Metformin | Sigma-Aldrich | Water | 3 mM |
| Malonate | Sigma-Aldrich | Water | 5 mM |
| Etomoxir | Sigma-Aldrich | Water | 20-50 mM |
| Doxycycline | Sigma-Aldrich | Water | 2 mg/ml |
| GW501516 | MedChemExpress | DMSO | 1, 5, 10 mM |
| GW0742 | MedChemExpress | DMSO | 1, 5, 10 mM |
| L-165 | MedChemExpress | DMSO | 1, 5, 10 mM |
| DG172 | Tocris Bioscience | DMSO | 1 mM |
| Mycro3 | Aobious | DMSO | 25 mM |

**Table S2.** Main chemicals used for *in vitro* and *in vivo* treatments. Information about compound, company, solvent used for stock preparation and working concentration is listed.
