# Supplementary material for: PPARδ Orchestrates a Prometastatic Metabolic Response to Microenvironmental Cues in Pancreatic Cancer
Source: Cancer Res. 2025 Jul 3;85(17):3275–91. doi: 10.1158/0008-5472.CAN-24-3475 (PMC12402788; doi:10.1158/0008-5472.CAN-24-3475)
Supplement: Table S3 — Plasmids used in this study [file can-24-3475_table_s3_suppst3.docx]

| **Target** | **Construct** | **Company** | **Reference** |
| --- | --- | --- | --- |
| PPAR-δ | shRNA, inducible | Transomic Technologies | TLHSU2300-5467 |
| MYC | shRNA, inducible | Transomic Technologies | TLHSU2300-4609 |
| PGC-1α | Overexpression, inducible | Gift | ^58^ |
| PPAR-δ | Overexpression, inducible | Gift | ^43^ |
| PGC-1α promoter | Reporter | Active motif | S722424 |
| HBM-luc | Reporter | Gift | ^63^  (Addgene_35155) |

**Table S3.** Plasmids used in this study. Information about target gene, type of construct, company and reference is listed.
