# Supplementary material for: PPARδ Orchestrates a Prometastatic Metabolic Response to Microenvironmental Cues in Pancreatic Cancer
Source: Cancer Res. 2025 Jul 3;85(17):3275–91. doi: 10.1158/0008-5472.CAN-24-3475 (PMC12402788; doi:10.1158/0008-5472.CAN-24-3475)
Supplement: Table S4 — Primers for rt-qPCR used in this study [file can-24-3475_table_s4_suppst4.docx]

| **Gene** | **Forward Primer** | **Reverse Primer** |
| --- | --- | --- |
| *HPRT1* | TGACCTTGATTTATTTTGCATACC | CGAGCAAGACGTTCAGTCCT |
| *LOXL2* | GGCACCGTGTGCGATGACGA | GCTGCAAGGGTCGCCTCGTT |
| *MYC* | CCCGCTTCTCTGAAAGGCTCTC | CTCTGCTGCTGCTGCTGGTAG |
| *PPARGC1A* | TGACTGGCGTCATTCAGGAG | CCAGAGCAGCACACTCGAT |
| *PPARA* | CTGGAAGCTTTGGCTTTACG | ACCAGCTTGAGTCGAATCGT |
| *PPARD* | CTCTATCGTCAACAAGGACG | GTCTTCTTGATCCGCTGCAT |
| *PPARG* | GACCTGAAACTTCAAGAGTAC | TGAGGCTTATTGTAGAGCTGAGTC |
| *SNAI2* | ATGCCGCGCTCCTTCCT | TGTGTCCAGTTCGCT |
| *SNAI1* | GCTCCTTCGTCCTTCTCCTC | TGACATCTGAGTGGGTCTGG |
| *VIM* | GACAATGCGTCTCTGGCACGTCTT | TCCTCCGCCTCCTGCAGGTTCTT |
| *ZEB1* | GTTGATGAATGCGAGTCAGATGC | CTGGTCCTCTTCAGGTGCC |
| *GFP* | GCAAAGACCCCAACGAGAAG | TCACGAACTCCAGCAGGACC |
| *ABCA4* | AGAATAACCGGACGCTGCTC | TCACCAAACCGGGCATAGAC |
| *APOA1* | TGCCCACTCTATTTGCCCAG | CTCACTGGTCCTGGCAATGT |
| *ETFDH* | GGACAGTCCTCCTGTTGTGT | TTCCATGTTCACTCCCAGGCA |
| *FABP4* | TGGGCCAGGAATTTGACGAA | CACATGTACCAGGACACCCC |
| *MLYCD* | GTCCGGGAAATGAATGGGGT | GCCAGGTAACCCGTTCTAGG |
| *SLC27A4* | GGCTCAGGGGCCAATAAACT | ACAGATGAGGCGGGTCAATG |
| *HSPD1* | CCTGCACTCTGTCCCTCACTC | GGTCTCATCTGGCGAAAGACT |
| *TXNIP* | CAACTTGCTGCCCGACAAAA | TGGGTGGCATGCAAGGTATT |
| *APOE* | GTTGCTGGTCACATTCCTGG | GCAGGTAATCCCAAAAGCGAC |
| *DGAT1* | TCGCCTGCAGGATTCTTTAT | GCATCACCACACACCAGTTC |
| *GAPDH* | TCCTGTTCGACAGTCAGCCGC | ACGACCAAATCCGTTGACTCCG |
| *Gapdh* | TGCACCACCAACTGCTTAGC | TCTTCTGGGTGGCAGTGATG |
| *ZsGreen* | CGGGGATGATCTTTTCGCAG | CTGCATGTACCACGAGTCCA |

**Table S4.** Primers for rt-qPCR used in this study. Information about target gene, forward sequence and reverse sequence is listed.
