# Supplementary material for: PPARδ Orchestrates a Prometastatic Metabolic Response to Microenvironmental Cues in Pancreatic Cancer
Source: Cancer Res. 2025 Jul 3;85(17):3275–91. doi: 10.1158/0008-5472.CAN-24-3475 (PMC12402788; doi:10.1158/0008-5472.CAN-24-3475)
Supplement: Table S5 — Antibodies used in this study [file can-24-3475_table_s5_suppst5.docx]

| **Protein** | **Company** | **Reference** | **RRID** | **Clone** |
| --- | --- | --- | --- | --- |
| **Western Blot** | | | | |
| PPAR-α | Santa Cruz Biotechnology | sc-398394 | AB_2885073 | H-2 |
| PPAR-δ | Santa Cruz Biotechnology | sc-74517 | AB_1128604 | F-10 |
| PPAR-γ | Santa Cruz Biotechnology | sc-7273 | AB_628115 | E-8 |
| c-MYC | Cell Signaling Technology | 9402S | AB_2151827 |  |
| PGC-1α | Santa Cruz Biotechnology | sc-13067 | AB_2166218 | H300 |
| Vinculin | Sigma Aldrich | SAB4200080 | AB_10604160 | V-284 |
| β-Actin | Sigma Aldrich | A2228 | AB_476697 | AC-74 |
| anti-mouse | Thermo Fisher Scientific | A16160 | AB_2534831 |  |
| anti-rabbit | Thermo Fisher Scientific | A16096 | AB_2534770 |  |
| **Immunohistochemistry** | | | | |
| c-MYC | Abcam | ab32072 | AB_731658 | Y69 |
| CK-19 | DAKO | IR615/IS615 |  | RCK108 |
| HIF-1α | LSBio | LS-B495 | AB_2232956 |  |
| PPAR-δ | Abcam | ab23673 | AB_2165902 |  |
| Vimentin | DAKO | IR630 |  | V9 |
| Anti-mouse | Vector Labs | MP-7452 | AB_2744550 |  |
| Anti-rabbit | Vector Labs | MP-7451 | AB_2631198 |  |
| **CUT&Tag** | | | | |
| PPAR-δ | Cell signaling | 74076 | AB_3661825 |  |
| c-MYC | Santa Cruz Biotechnology | sc-40 X | AB_2857941 |  |
| anti-rabbit | Abcam | ab6702 | AB_956012 |  |
| anti-mouse | Abcam | ab6708 | AB_956005 |  |

**Table S5.** Antibodies used in this study. Information about target protein, company, reference, research resource identifiers (RRID) and clone is listed and specified for each technique.
