# Supplementary material for: PPARδ Orchestrates a Prometastatic Metabolic Response to Microenvironmental Cues in Pancreatic Cancer
Source: Cancer Res. 2025 Jul 3;85(17):3275–91. doi: 10.1158/0008-5472.CAN-24-3475 (PMC12402788; doi:10.1158/0008-5472.CAN-24-3475)
Supplement: Table S6 — Pharmacological targeting of PPAR-δ modulates metastasis in vivo [file can-24-3475_table_s6_suppst6.docx]

| **Parameters/Group** | **Control** | **GW0742** | **GSK0660** |
| --- | --- | --- | --- |
| **Macrometastases (%)** | 75 (6/8) | 78 (7/9) | 60 (6/10) |
| **Total CK area (mm2)** | 0.38 ± 0.13 | 1.31 ± 0.48* | 0.15 ± 0.09* |

**Table S6.** Pharmacological targeting of PPAR-δ modulates metastasis *in vivo*. Table related to the data included in Figure S14. Quantification of macrometastases (percentage of livers with at least one macroscopic metastasis) and micrometastases (total area stained for CK-19) in livers from mice treated as indicated. *p<0.05.
