# Supplementary material for: PPARδ Orchestrates a Prometastatic Metabolic Response to Microenvironmental Cues in Pancreatic Cancer
Source: Cancer Res. 2025 Jul 3;85(17):3275–91. doi: 10.1158/0008-5472.CAN-24-3475 (PMC12402788; doi:10.1158/0008-5472.CAN-24-3475)
Supplement: Figure S1 — Different stimuli promoting mitochondrial energy deprivation or coculture with stromal cells the expression of epithelial-to-mesenchymal transition genes in PDAC cells [file can-24-3475_figure_s1_suppsf1.pptx]

## Slide 1
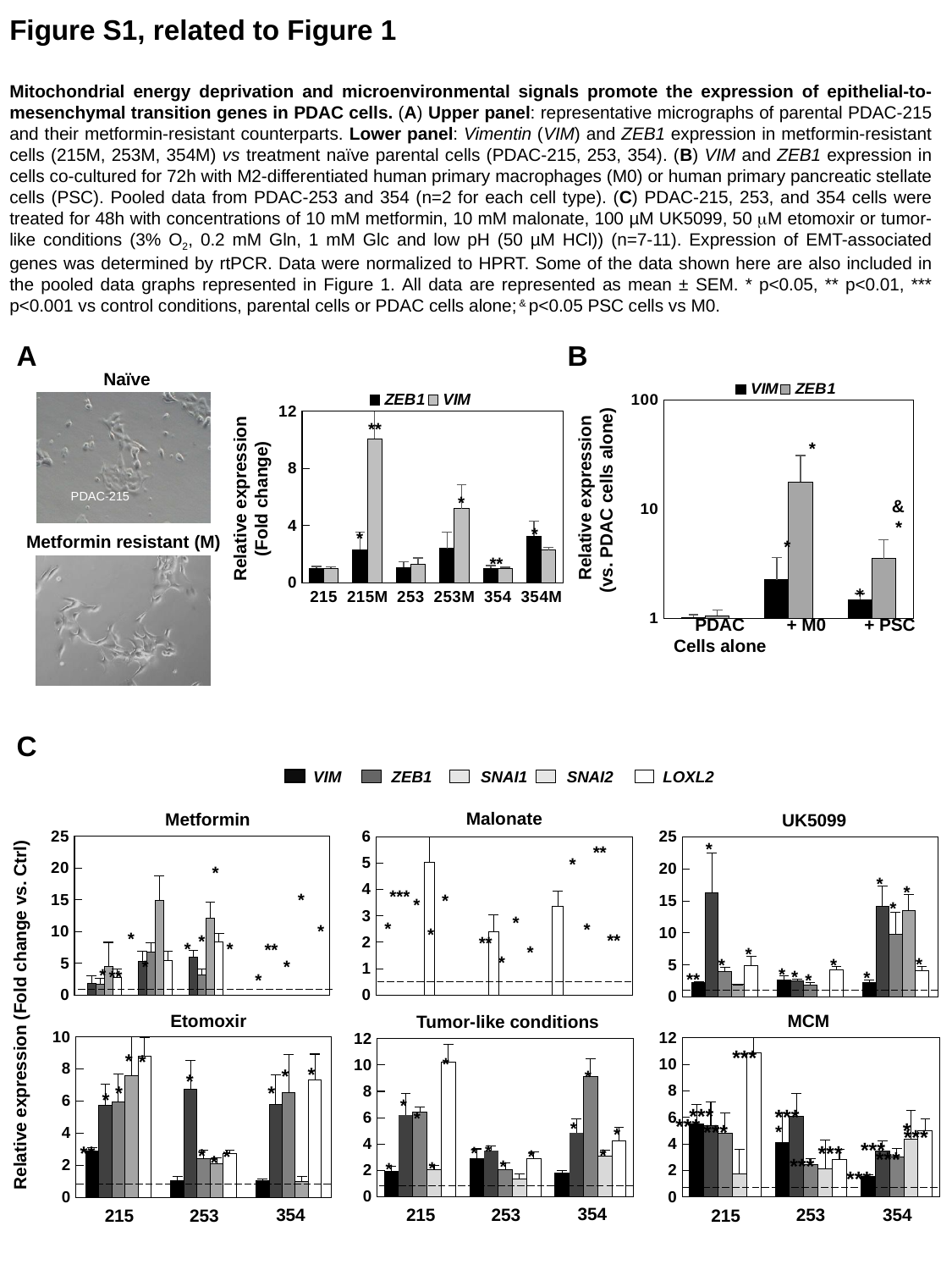

Figure S1, related to Figure 1
Mitochondrial energy deprivation and microenvironmental signals promote the expression of epithelial-to-mesenchymal transition genes in PDAC cells. (A) Upper panel: representative micrographs of parental PDAC-215 and their metformin-resistant counterparts. Lower panel: Vimentin (VIM) and ZEB1 expression in metformin-resistant cells (215M, 253M, 354M) vs treatment naïve parental cells (PDAC-215, 253, 354). (B) VIM and ZEB1 expression in cells co-cultured for 72h with M2-differentiated human primary macrophages (M0) or human primary pancreatic stellate cells (PSC). Pooled data from PDAC-253 and 354 (n=2 for each cell type). (C) PDAC-215, 253, and 354 cells were treated for 48h with concentrations of 10 mM metformin, 10 mM malonate, 100 µM UK5099, 50 mM etomoxir or tumor-like conditions (3% O2, 0.2 mM Gln, 1 mM Glc and low pH (50 µM HCl)) (n=7-11). Expression of EMT-associated genes was determined by rtPCR. Data were normalized to HPRT. Some of the data shown here are also included in the pooled data graphs represented in Figure 1. All data are represented as mean ± SEM. * p<0.05, ** p<0.01, *** p<0.001 vs control conditions, parental cells or PDAC cells alone; & p<0.05 PSC cells vs M0.
A
B
### Chart
| Category | ZEB1 | VIM |
|---|---|---|
| 215 | 1.011106767749094 | 1.02577648226955 |
| 215M | 2.320887730160348 | 10.07127625877262 |
| 253 | 1.068352387462742 | 1.30129245821623 |
| 253M | 2.409991875943628 | 5.197290554407536 |
| 354 | 1.015642380792242 | 1.013135850090928 |
| 354M | 3.266126112722904 | 2.296054063777292 |**
*
*
*
**
Relative expression
(Fold change)
Naïve
Metformin resistant (M)
### Chart
| Category | VIM | ZEB1 |
|---|---|---|
| single | 1.013554517039612 | 1.05136003492372 |
| Co-cult M0 | 2.240721996400303 | 17.78456884711611 |
| co-cult CAF | 1.473252125384135 | 3.517642154645923 |*
&
*
*
*
PDAC
Cells alone
+ M0
+ PSC
Relative expression
(vs. PDAC cells alone)
PDAC-215
C
VIM
ZEB1
SNAI1
SNAI2
LOXL2
### Chart
| Category | VIM | ZEB1 | SLUG | SNAIL | LOXL2 |
|---|---|---|---|---|---|
| 100 | 2.25735428708002 | 16.269835257375643 | 3.922508035051248 | 1.9171977820055819 | 4.916901450007643 |
| 100 | 2.6980823764544675 | 2.5005571381823777 | 1.8786702331975131 | 0.0 | 4.231174207696885 |
| 100 | 2.2167196952674124 | 14.184520195426083 | 9.765192645256596 | 13.501760081959068 | 4.045324405336781 |UK5099
*
*
*
*
*
*
*
*
*
*
*
**
*
### Chart
| Category | VIM | ZEB1 | SLUG | SNAIL | LOXL2 |
|---|---|---|---|---|---|
| Met 10 | 0.9800998714506446 | 1.82918392555124 | 1.7050253313648258 | 4.540239598239014 | 2.8195712802751194 |
| Met 10 | 1.4563236978431455 | 5.263809066679028 | 6.753408552957245 | 14.956994997185639 | 5.416937393740983 |
| Met 10 | 2.0993815282501638 | 6.015423561199107 | 3.119452160541911 | 12.075499758916317 | 8.32147250124955 |Metformin
*
*
*
*
*
*
**
*
*
*
**
*
### Chart
| Category | VIM | ZEB1 | SLUG | SNAIL | LOXL2 |
|---|---|---|---|---|---|
| Mal 10 | 3.6111185389377196 | 6.714504778233138 | 4.370851609071522 | 3.33213571030005 | 5.034445038333504 |
| Mal 10 | 0.6773873262704234 | 3.6679951525811108 | 1.847067010665154 | 3.754203914684203 | 2.3919276373749434 |
| Mal 10 | 1.4113242341909382 | 7.630252443900666 | 3.9010983697384347 | 10.297071797600381 | 3.360592037572555 |Malonate
**
*
***
*
*
*
*
*
*
*
**
**
*
*
Relative expression (Fold change vs. Ctrl)
Etomoxir
### Chart
| Category | VIM | ZEB1 | SLUG | SNAIL | LOXL2 |
|---|---|---|---|---|---|
| 50 | 2.9031036504960004 | 5.725683735233189 | 5.972530804580138 | 7.595812684798358 | 8.781995644 |
| 50 | 1.0310703034668802 | 6.721351316650545 | 2.422182908643334 | 2.078237068271131 | 2.7055916945439797 |
| 50 | 1.0446379676509139 | 5.807263866081647 | 6.552348568366097 | 0.969190580520582 | 7.3480605421463485 |*
*
*
*
*
*
*
*
**
*
*
*
354
253
215
MCM
### Chart
| Category | | | SLUG | SNAIL | |
|---|---|---|---|---|---|
| 215 | 5.533478209872815 | 5.396507549437868 | 4.820867272470874 | 1.7133663037946916 | 10.878613987106919 |
| 253 | 4.09469435945102 | 6.069236256383518 | 2.444745289572324 | 2.1318104916994143 | 2.854005082352753 |
| 354 | 1.5186432554894387 | 3.467517976124247 | 2.996888732023946 | 4.33726836153958 | 4.97215834808567 |***
***
***
***
*
***
*
***
***
***
***
***
***
354
253
215
Tumor-like conditions
### Chart
| Category | | | SLUG | SNAIL | LOXL2 |
|---|---|---|---|---|---|
| | 1.9306816860908702 | 6.184015209647385 | 6.393007965035597 | 2.0427312933913004 | 10.223511072012432 |
| T-like | 2.8827031267020176 | 3.4975467601588868 | 2.0269841294785262 | 1.3132429224110727 | 2.87729180700822 |*
*
*
*
*
*
*
*
*
*
*
*
*
354
253
215
