# Supplementary material for: PPARδ Orchestrates a Prometastatic Metabolic Response to Microenvironmental Cues in Pancreatic Cancer
Source: Cancer Res. 2025 Jul 3;85(17):3275–91. doi: 10.1158/0008-5472.CAN-24-3475 (PMC12402788; doi:10.1158/0008-5472.CAN-24-3475)
Supplement: Figure S2 — Incubation with a non-toxic dose of etomoxir and macrophage-conditioned medium does not affect CD133 expression or self-renewal of PDAC cells [file can-24-3475_figure_s2_suppsf2.pptx]

## Slide 1
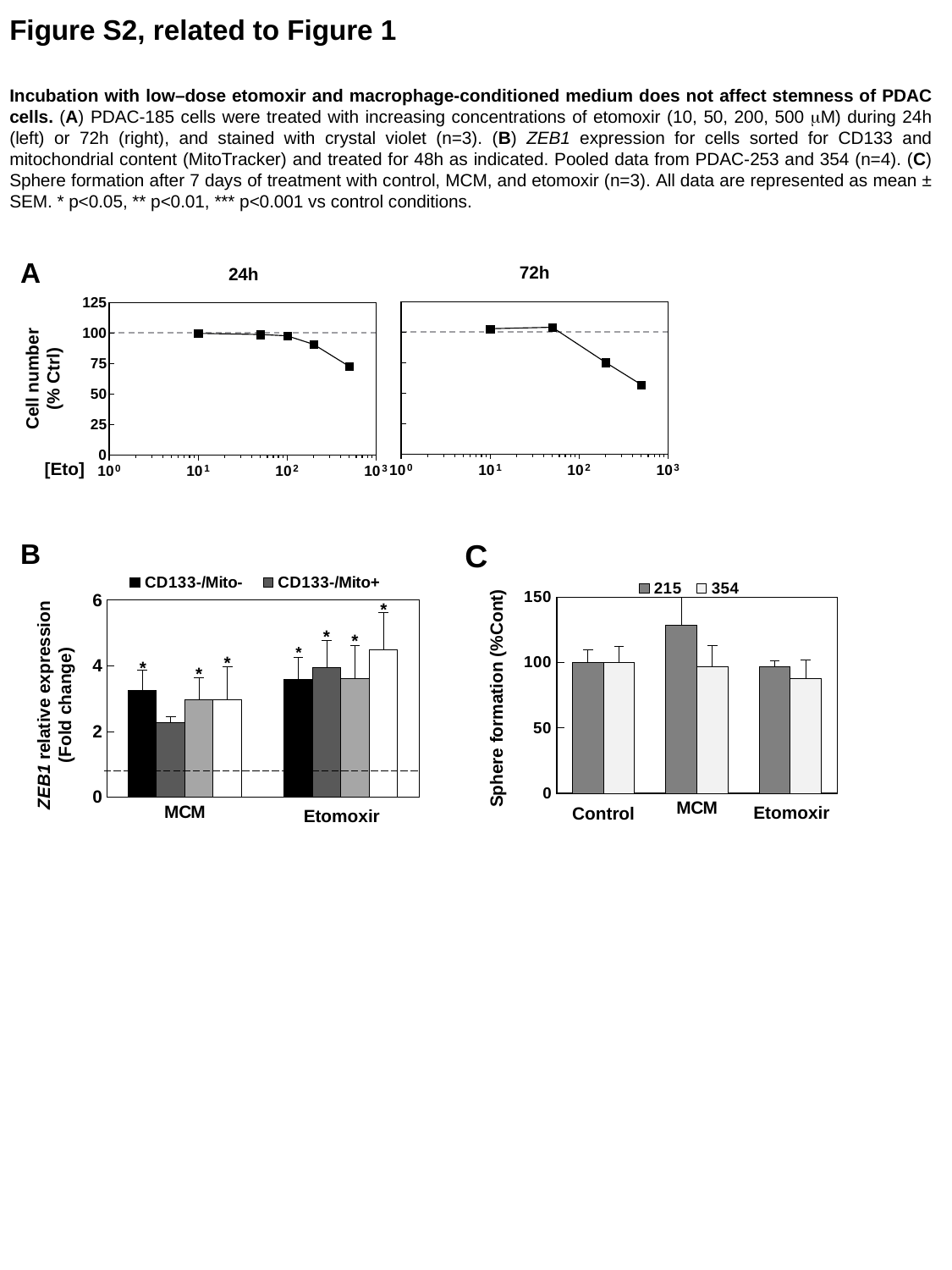

Figure S2, related to Figure 1
Incubation with low–dose etomoxir and macrophage-conditioned medium does not affect stemness of PDAC cells. (A) PDAC-185 cells were treated with increasing concentrations of etomoxir (10, 50, 200, 500 mM) during 24h (left) or 72h (right), and stained with crystal violet (n=3). (B) ZEB1 expression for cells sorted for CD133 and mitochondrial content (MitoTracker) and treated for 48h as indicated. Pooled data from PDAC-253 and 354 (n=4). (C) Sphere formation after 7 days of treatment with control, MCM, and etomoxir (n=3). All data are represented as mean ± SEM. * p<0.05, ** p<0.01, *** p<0.001 vs control conditions.
A
72h
24h
Cell number
(% Ctrl)
[Eto]
B
C
### Chart
| Category | CD133-/Mito- | CD133-/Mito+ | CD133+/Mito- | CD133+/Mito+ |
|---|---|---|---|---|
| MCM | 3.25690765629508 | 2.259919221635096 | 2.963600348645875 | 2.955228559998376 |
| eto | 3.59558418372768 | 3.951097264664537 | 3.611323281559474 | 4.481134873128521 |ZEB1 relative expression
(Fold change)
Etomoxir
*
*
*
*
*
*
*
### Chart
| Category | 215 | |
|---|---|---|
| Cont | 100.0 | 100.0 |
| MCM | 128.4478338496027 | 96.42844876359507 |
| Eto | 96.4509394572025 | 87.82608695652102 |Sphere formation (%Cont)
Etomoxir
Control
