# Supplementary material for: PPARδ Orchestrates a Prometastatic Metabolic Response to Microenvironmental Cues in Pancreatic Cancer
Source: Cancer Res. 2025 Jul 3;85(17):3275–91. doi: 10.1158/0008-5472.CAN-24-3475 (PMC12402788; doi:10.1158/0008-5472.CAN-24-3475)
Supplement: Figure S3 — Etomoxir or stromal signals alter different metabolic parameters in PDAC cells [file can-24-3475_figure_s3_suppsf3.pptx]

## Slide 1
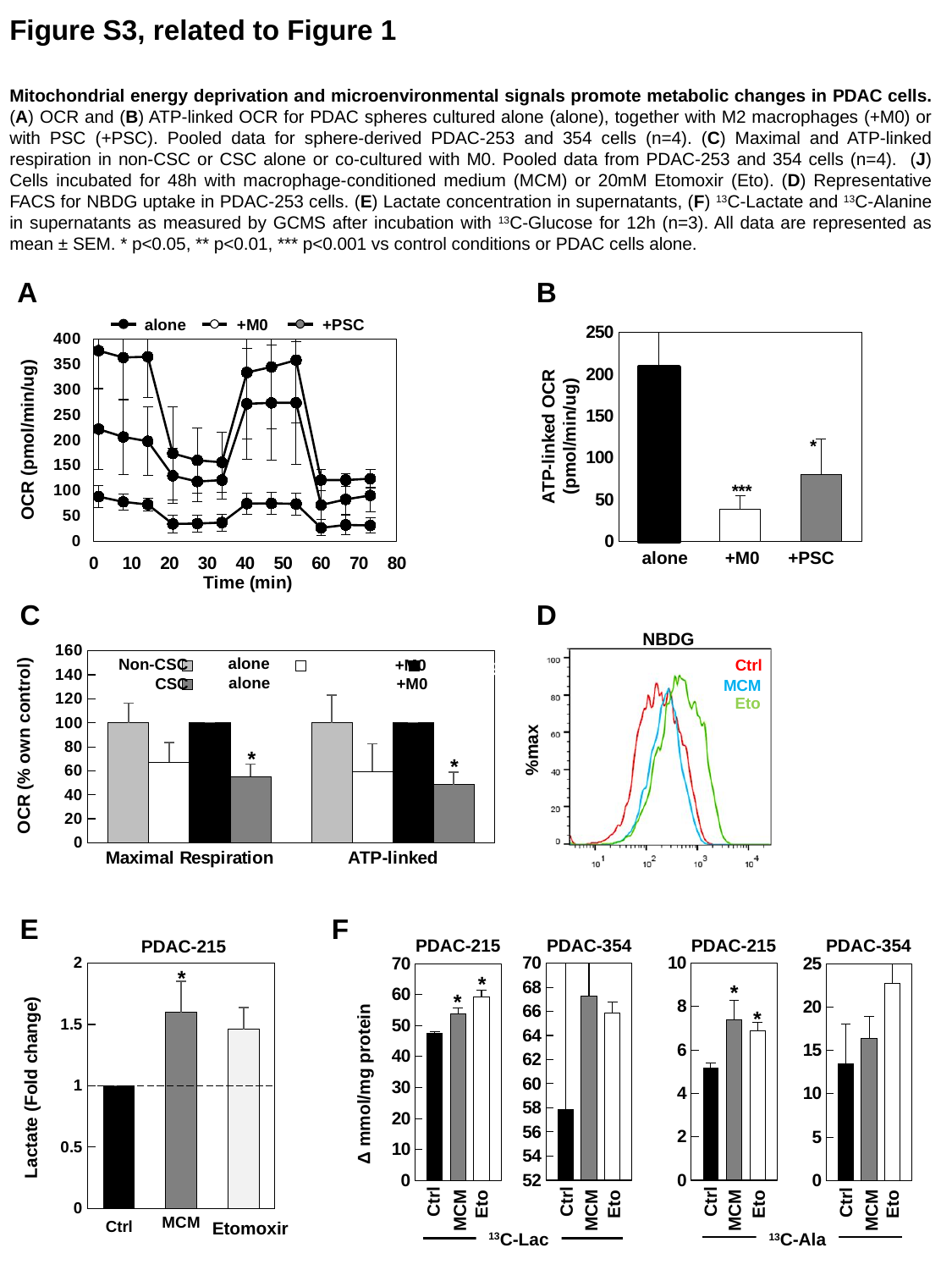

Figure S3, related to Figure 1
Mitochondrial energy deprivation and microenvironmental signals promote metabolic changes in PDAC cells. (A) OCR and (B) ATP-linked OCR for PDAC spheres cultured alone (alone), together with M2 macrophages (+M0) or with PSC (+PSC). Pooled data for sphere-derived PDAC-253 and 354 cells (n=4). (C) Maximal and ATP-linked respiration in non-CSC or CSC alone or co-cultured with M0. Pooled data from PDAC-253 and 354 cells (n=4). (J) Cells incubated for 48h with macrophage-conditioned medium (MCM) or 20mM Etomoxir (Eto). (D) Representative FACS for NBDG uptake in PDAC-253 cells. (E) Lactate concentration in supernatants, (F) 13C-Lactate and 13C-Alanine in supernatants as measured by GCMS after incubation with 13C-Glucose for 12h (n=3). All data are represented as mean ± SEM. * p<0.05, ** p<0.01, *** p<0.001 vs control conditions or PDAC cells alone.
A
B
### Chart
| Category | single | co-cult M0 | co-cult CAF | Unassigned | Unselected |
|---|---|---|---|---|---|
alone
+PSC
+M0
OCR (pmol/min/ug)
### Chart
| Category | ATP Production |
|---|---|
| single | 209.0853424072266 |
| co-cult M0 | 38.41434860229489 |
| co-cult CAF | 79.81023406982423 |*
***
ATP-linked OCR
(pmol/min/ug)
alone
+M0
+PSC
C
D
NBDG
Ctrl
MCM
Eto
%max
### Chart
| Category | Adh single | Adh co-cult | Sph single | Sph co-cult |
|---|---|---|---|---|
| Maximal Respiration | 100.0 | 67.05238604152842 | 100.0 | 55.05517826080765 |
| ATP-linked | 100.0 | 59.38447754234265 | 100.0 | 48.32786388863492 |*
*
*
alone
Non-CSC
+M0
alone
CSC
+M0
OCR (% own control)
### Chart
| Category | Ctrl | MCM | Eto |
|---|---|---|---|
| 13C Ala | 5.167247133427112 | 7.39132738566288 | 6.889915917419636 |
### Chart
| Category | Ctrl | MCM | Eto |
|---|---|---|---|
| 13C Lac | 47.55199521357798 | 53.7753253983386 | 59.14320770105482 |
### Chart
| Category | Ctrl | MCM | Eto |
|---|---|---|---|
| 13C Ala | 13.50402885560374 | 16.39137386802168 | 22.68456429262895 |PDAC-215
PDAC-354
PDAC-215
PDAC-354
### Chart
| Category | Ctrl | MCM | Eto |
|---|---|---|---|
| 13C Lac | 57.86275140619421 | 67.27611859188069 | 65.86144054262347 |*
*
*
*
Δ mmol/mg protein
Ctrl
Ctrl
Ctrl
Ctrl
Eto
Eto
Eto
Eto
MCM
MCM
MCM
MCM
13C-Lac
13C-Ala
E
F
PDAC-215
### Chart
| Category | |
|---|---|
| Cont | 1.0 |
| MCM | 1.598283138715312 |
| eto | 1.461349087019897 |*
Lactate (Fold change)
Ctrl
Etomoxir
