# Supplementary material for: PPARδ Orchestrates a Prometastatic Metabolic Response to Microenvironmental Cues in Pancreatic Cancer
Source: Cancer Res. 2025 Jul 3;85(17):3275–91. doi: 10.1158/0008-5472.CAN-24-3475 (PMC12402788; doi:10.1158/0008-5472.CAN-24-3475)
Supplement: Figure S4 — Incubation with etomoxir and MCM induce epithelial-to-mesenchymal transition at the single-cell level [file can-24-3475_figure_s4_suppsf4.pptx]

## Slide 1
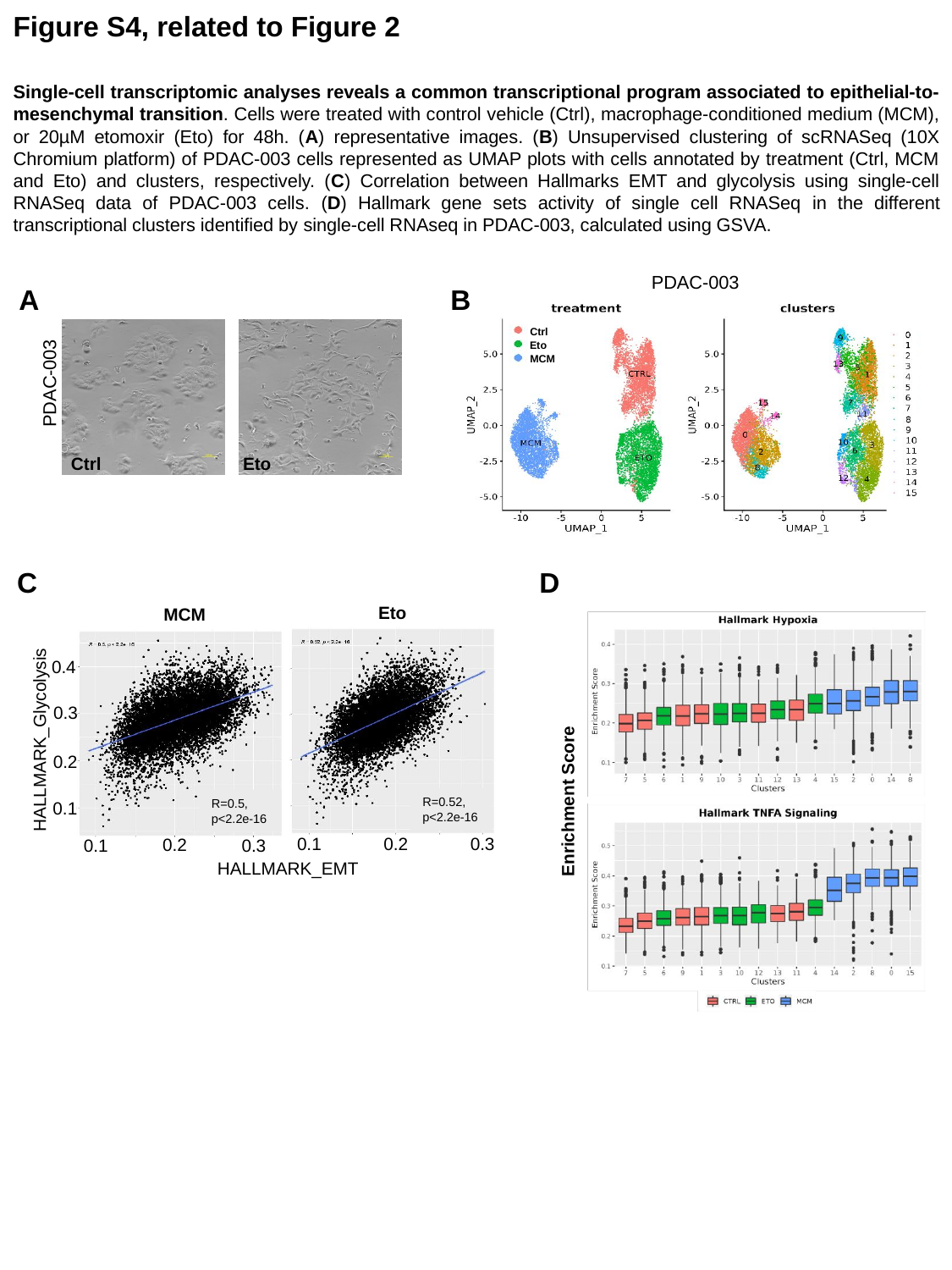

Figure S4, related to Figure 2
Single-cell transcriptomic analyses reveals a common transcriptional program associated to epithelial-to-mesenchymal transition. Cells were treated with control vehicle (Ctrl), macrophage-conditioned medium (MCM), or 20µM etomoxir (Eto) for 48h. (A) representative images. (B) Unsupervised clustering of scRNASeq (10X Chromium platform) of PDAC-003 cells represented as UMAP plots with cells annotated by treatment (Ctrl, MCM and Eto) and clusters, respectively. (C) Correlation between Hallmarks EMT and glycolysis using single-cell RNASeq data of PDAC-003 cells. (D) Hallmark gene sets activity of single cell RNASeq in the different transcriptional clusters identified by single-cell RNAseq in PDAC-003, calculated using GSVA.
PDAC-003
Ctrl
Eto
MCM
A
B
PDAC-003
Ctrl
Eto
C
D
Enrichment Score
Eto
MCM
0.4
0.3
HALLMARK_Glycolysis
0.2
R=0.52, p<2.2e-16
R=0.5, p<2.2e-16
0.1
0.1
0.2
0.3
0.2
0.1
0.3
HALLMARK_EMT
