# Supplementary material for: PPARδ Orchestrates a Prometastatic Metabolic Response to Microenvironmental Cues in Pancreatic Cancer
Source: Cancer Res. 2025 Jul 3;85(17):3275–91. doi: 10.1158/0008-5472.CAN-24-3475 (PMC12402788; doi:10.1158/0008-5472.CAN-24-3475)
Supplement: Figure S5 — EMT induction by etomoxir and MCM in bulk transcriptomics [file can-24-3475_figure_s5_suppsf5.pptx]

## Slide 1
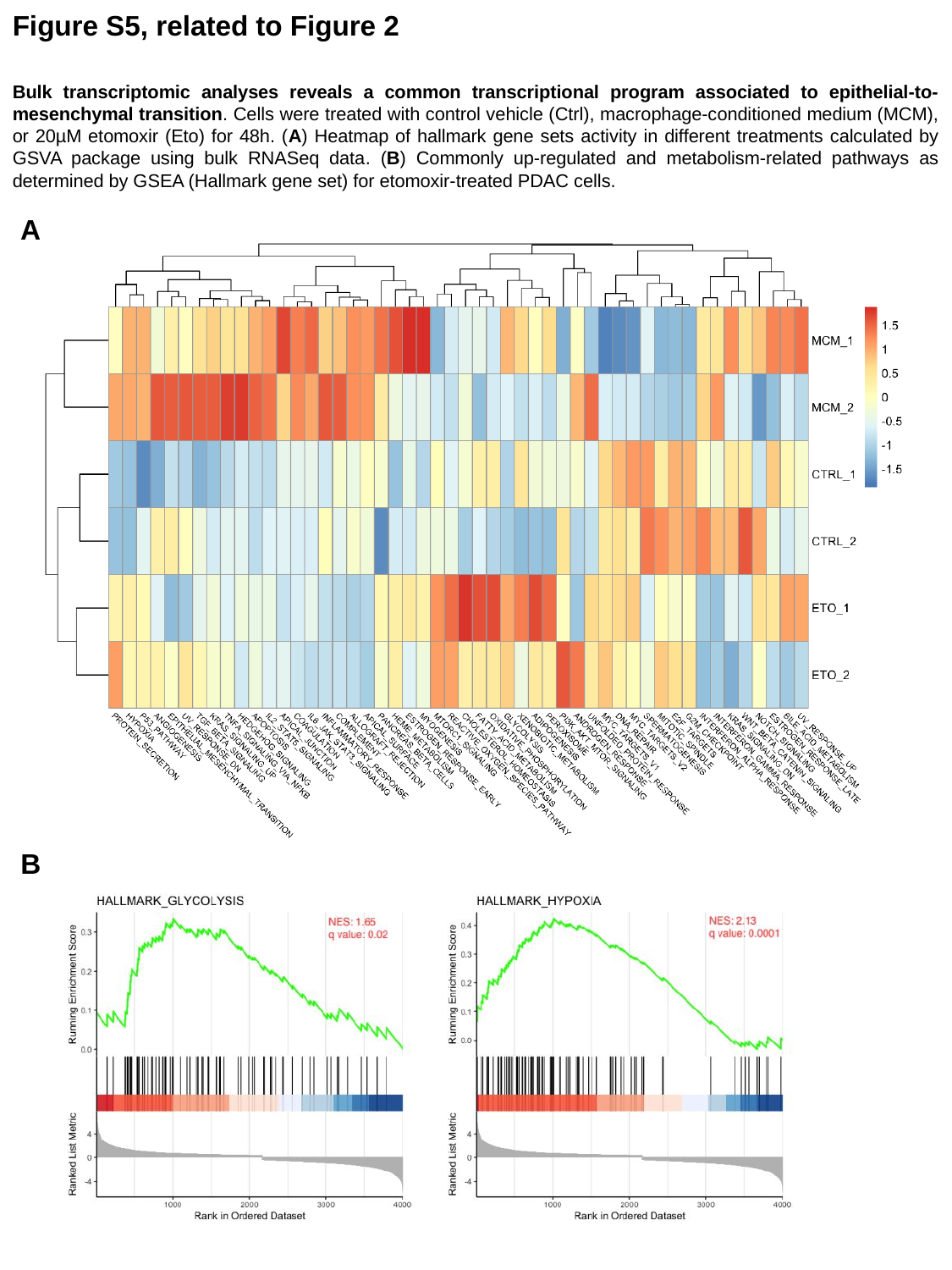

Figure S5, related to Figure 2
Bulk transcriptomic analyses reveals a common transcriptional program associated to epithelial-to-mesenchymal transition. Cells were treated with control vehicle (Ctrl), macrophage-conditioned medium (MCM), or 20µM etomoxir (Eto) for 48h. (A) Heatmap of hallmark gene sets activity in different treatments calculated by GSVA package using bulk RNASeq data. (B) Commonly up-regulated and metabolism-related pathways as determined by GSEA (Hallmark gene set) for etomoxir-treated PDAC cells.
A
B
