# Supplementary material for: PPARδ Orchestrates a Prometastatic Metabolic Response to Microenvironmental Cues in Pancreatic Cancer
Source: Cancer Res. 2025 Jul 3;85(17):3275–91. doi: 10.1158/0008-5472.CAN-24-3475 (PMC12402788; doi:10.1158/0008-5472.CAN-24-3475)
Supplement: Figure S6 — Different expression analyses performed for PPARA and PPARG in the TCGA database [file can-24-3475_figure_s6_suppsf6.pptx]

## Slide 1
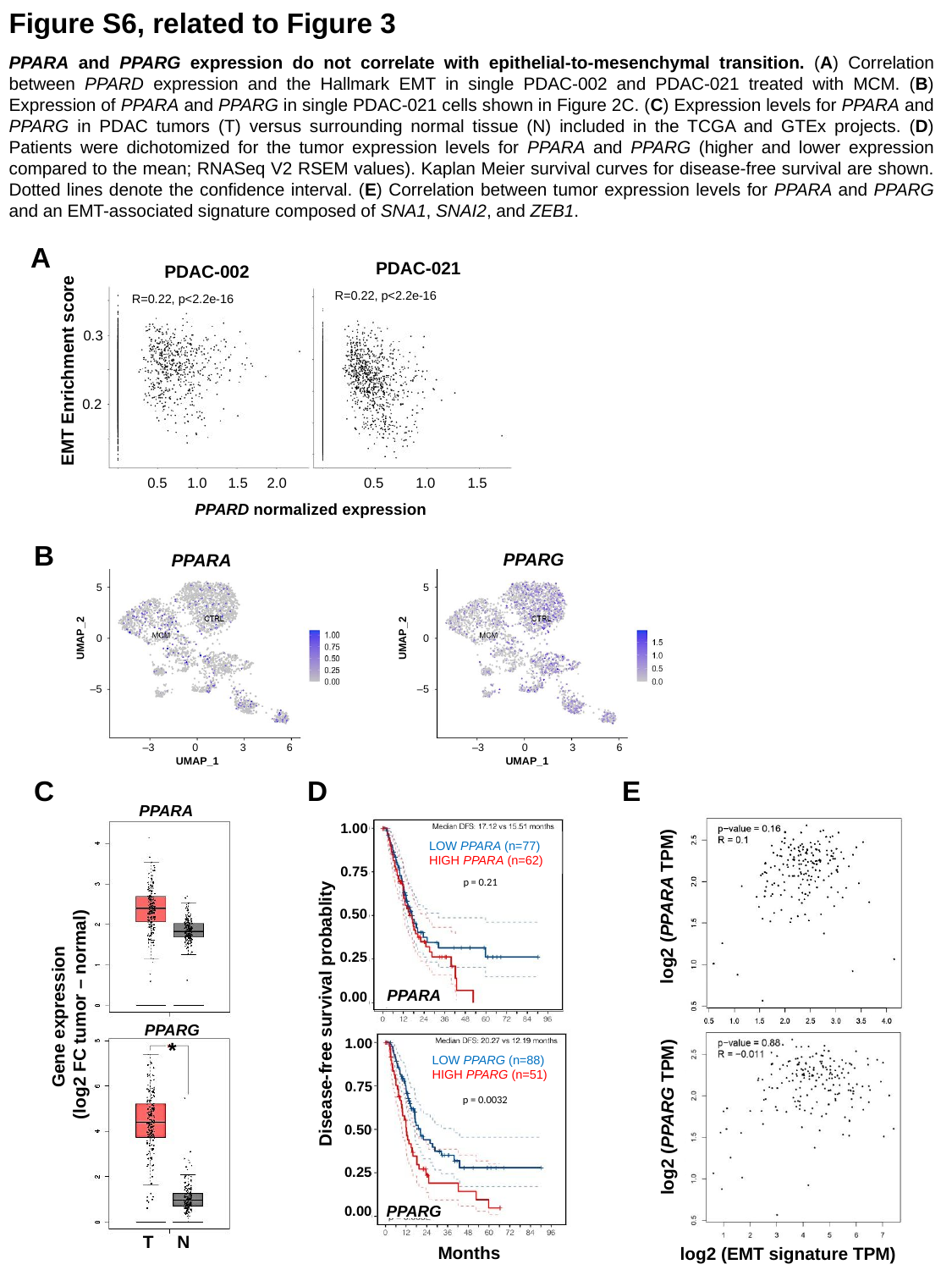

Figure S6, related to Figure 3
PPARA and PPARG expression do not correlate with epithelial-to-mesenchymal transition. (A) Correlation between PPARD expression and the Hallmark EMT in single PDAC-002 and PDAC-021 treated with MCM. (B) Expression of PPARA and PPARG in single PDAC-021 cells shown in Figure 2C. (C) Expression levels for PPARA and PPARG in PDAC tumors (T) versus surrounding normal tissue (N) included in the TCGA and GTEx projects. (D) Patients were dichotomized for the tumor expression levels for PPARA and PPARG (higher and lower expression compared to the mean; RNASeq V2 RSEM values). Kaplan Meier survival curves for disease-free survival are shown. Dotted lines denote the confidence interval. (E) Correlation between tumor expression levels for PPARA and PPARG and an EMT-associated signature composed of SNA1, SNAI2, and ZEB1.
A
PDAC-021
PDAC-002
R=0.22, p<2.2e-16
R=0.22, p<2.2e-16
0.3
EMT Enrichment score
0.2
0.5
1.0
1.5
2.0
0.5
1.0
1.5
PPARD normalized expression
B
PPARG
PPARA
5
5
0
UMAP_2
0
UMAP_2
–5
–5
–3
0
3
6
–3
0
3
6
UMAP_1
UMAP_1
C
D
E
PPARA
log2 (PPARA TPM)
log2 (PPARG TPM)
log2 (EMT signature TPM)
1.00
LOW PPARA (n=77)
HIGH PPARA (n=62)
0.75
p = 0.21
0.50
0.25
PPARA
0.00
Disease-free survival probablity
1.00
LOW PPARG (n=88)
HIGH PPARG (n=51)
0.75
p = 0.0032
0.50
0.25
0.00
Months
Gene expression
(log2 FC tumor – normal)
PPARG
*
PPARG
T
N
