# Supplementary material for: PPARδ Orchestrates a Prometastatic Metabolic Response to Microenvironmental Cues in Pancreatic Cancer
Source: Cancer Res. 2025 Jul 3;85(17):3275–91. doi: 10.1158/0008-5472.CAN-24-3475 (PMC12402788; doi:10.1158/0008-5472.CAN-24-3475)
Supplement: Figure S7 — Expression of the PPAR family members and downstream targets after incubation with etomoxir and MCM [file can-24-3475_figure_s7_suppsf7.pptx]

## Slide 1
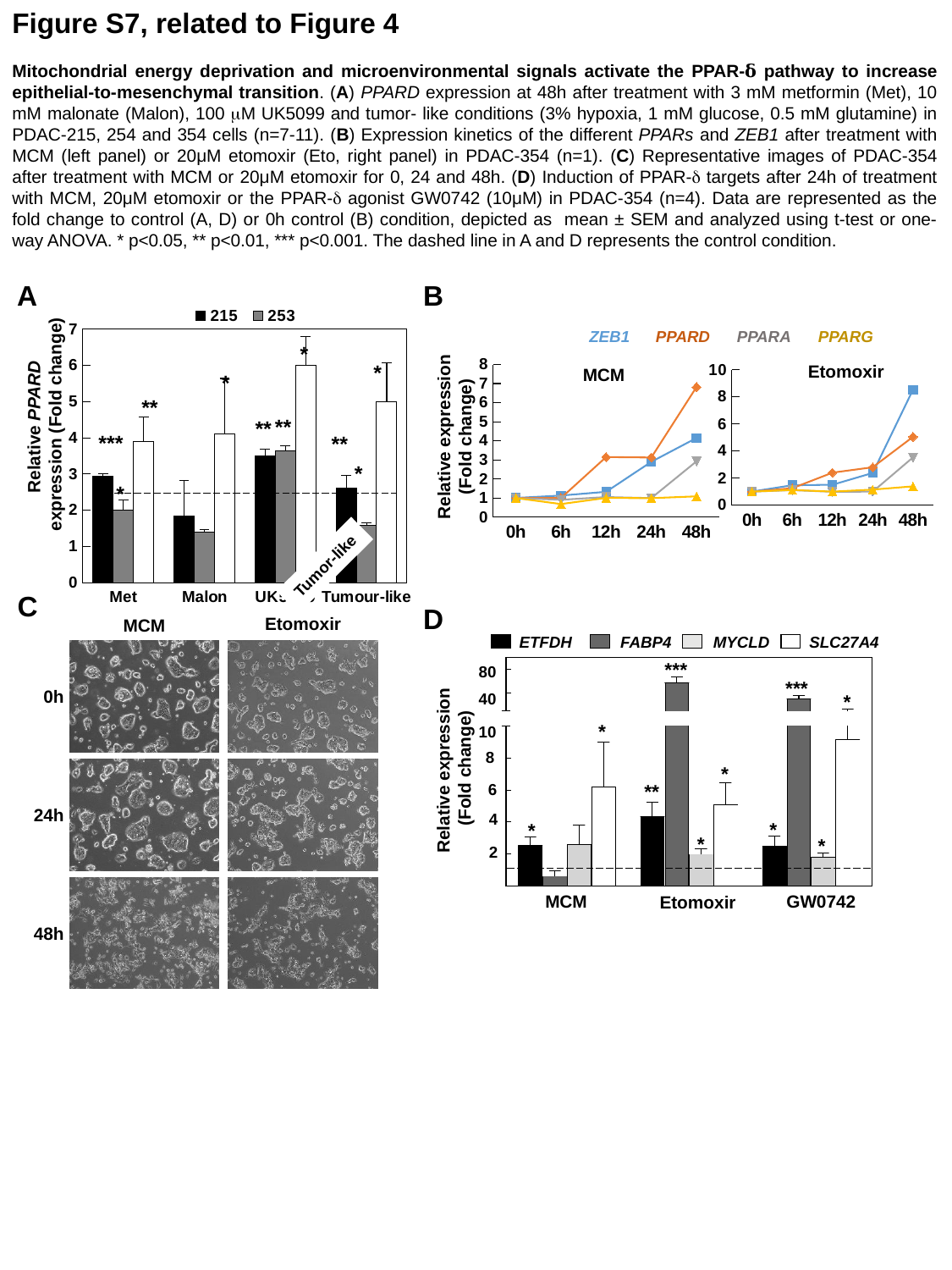

Figure S7, related to Figure 4
Mitochondrial energy deprivation and microenvironmental signals activate the PPAR-𝛅 pathway to increase epithelial-to-mesenchymal transition. (A) PPARD expression at 48h after treatment with 3 mM metformin (Met), 10 mM malonate (Malon), 100 mM UK5099 and tumor- like conditions (3% hypoxia, 1 mM glucose, 0.5 mM glutamine) in PDAC-215, 254 and 354 cells (n=7-11). (B) Expression kinetics of the different PPARs and ZEB1 after treatment with MCM (left panel) or 20μM etomoxir (Eto, right panel) in PDAC-354 (n=1). (C) Representative images of PDAC-354 after treatment with MCM or 20μM etomoxir for 0, 24 and 48h. (D) Induction of PPAR-d targets after 24h of treatment with MCM, 20μM etomoxir or the PPAR-d agonist GW0742 (10μM) in PDAC-354 (n=4). Data are represented as the fold change to control (A, D) or 0h control (B) condition, depicted as mean ± SEM and analyzed using t-test or one-way ANOVA. * p<0.05, ** p<0.01, *** p<0.001. The dashed line in A and D represents the control condition.
A
B
### Chart
| Category | 215 | 253 | 354 |
|---|---|---|---|
| Met | 2.9330221061889206 | 1.995588036893188 | 3.908896591979367 |
| Malon | 1.8408104025931191 | 1.3952428772812047 | 4.11715595261704 |
| UK5099 | 3.5065557397656835 | 3.630220361662987 | 5.999171388376223 |
| Tumour-like | 2.6010648983095073 | 1.5769368021918526 | 4.998478244999157 |*
*
*
**
Relative PPARD
expression (Fold change)
**
**
***
**
*
*
Tumor-like
ZEB1
PPARD
PPARA
PPARG
### Chart
| Category | ZEB1 | PPARD | PPARA | PPARG |
|---|---|---|---|---|
| 0h | 1.005698419043302 | 1.003452395752721 | 1.001528125445187 | 1.002891150013376 |
| 6h | 1.47204884165691 | 1.249809245867734 | 1.109248698996325 | 1.120061700629962 |
| 12h | 1.509075763463888 | 2.401714402377783 | 0.99101989506618 | 1.002945994895615 |
| 24h | 2.381026676039028 | 2.800834327224018 | 1.000006005943606 | 1.152315265690071 |
| 48h | 8.531153203688344 | 5.051858468091744 | 3.528720429854599 | 1.391522483275381 |
### Chart
| Category | ZEB1 | PPARD | PPARA | PPARG |
|---|---|---|---|---|
| 0h | 1.005698419043302 | 1.003452395752721 | 1.001528125445187 | 1.002891150013376 |
| 6h | 1.133843080563041 | 1.00312251531514 | 0.909157045918285 | 0.688280710015212 |
| 12h | 1.328565707299942 | 3.149788398376373 | 1.052828452128695 | 1.008416931136792 |
| 24h | 2.900069437779797 | 3.126195002046811 | 1.0000361790643 | 0.996349866700388 |
| 48h | 4.150196798393249 | 6.817573136391986 | 2.92310277862627 | 1.090293513742824 |Etomoxir
MCM
Relative expression
(Fold change)
C
D
Etomoxir
MCM
0h
24h
48h
ETFDH
FABP4
MYCLD
SLC27A4
***
80
***
40
*
*
10
Relative expression
(Fold change)
8
*
6
**
4
*
*
*
*
2
MCM
GW0742
Etomoxir
