# Supplementary material for: PPARδ Orchestrates a Prometastatic Metabolic Response to Microenvironmental Cues in Pancreatic Cancer
Source: Cancer Res. 2025 Jul 3;85(17):3275–91. doi: 10.1158/0008-5472.CAN-24-3475 (PMC12402788; doi:10.1158/0008-5472.CAN-24-3475)
Supplement: Figure S8 — EMT induction by PPAR-δ agonists, lipidomic changes and inhibition of EMT by downregulation of PPARD [file can-24-3475_figure_s8_suppsf8.pptx]

## Slide 1
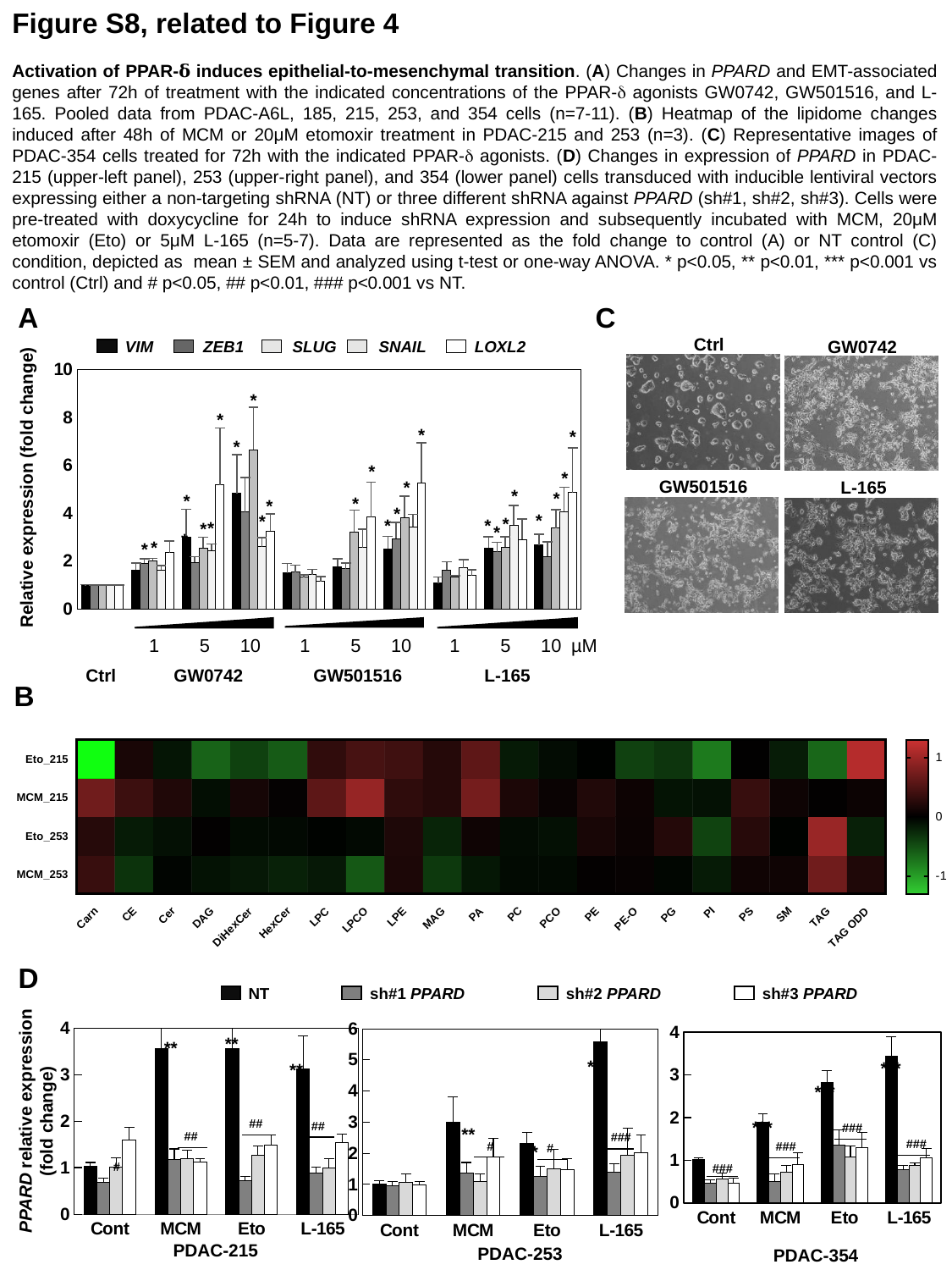

Figure S8, related to Figure 4
Activation of PPAR-𝛅 induces epithelial-to-mesenchymal transition. (A) Changes in PPARD and EMT-associated genes after 72h of treatment with the indicated concentrations of the PPAR-d agonists GW0742, GW501516, and L-165. Pooled data from PDAC-A6L, 185, 215, 253, and 354 cells (n=7-11). (B) Heatmap of the lipidome changes induced after 48h of MCM or 20μM etomoxir treatment in PDAC-215 and 253 (n=3). (C) Representative images of PDAC-354 cells treated for 72h with the indicated PPAR-d agonists. (D) Changes in expression of PPARD in PDAC-215 (upper-left panel), 253 (upper-right panel), and 354 (lower panel) cells transduced with inducible lentiviral vectors expressing either a non-targeting shRNA (NT) or three different shRNA against PPARD (sh#1, sh#2, sh#3). Cells were pre-treated with doxycycline for 24h to induce shRNA expression and subsequently incubated with MCM, 20μM etomoxir (Eto) or 5μM L-165 (n=5-7). Data are represented as the fold change to control (A) or NT control (C) condition, depicted as mean ± SEM and analyzed using t-test or one-way ANOVA. * p<0.05, ** p<0.01, *** p<0.001 vs control (Ctrl) and # p<0.05, ## p<0.01, ### p<0.001 vs NT.
A
C
### Chart
| Category | ZEB1 | SNAI1 | SLUG | LOXL2 | PPARD |
|---|---|---|---|---|---|
| CTL | 1.001766674205828 | 1.0 | 1.0 | 1.0 | 1.0 |
| GW0742 1uM | 1.612424540849071 | 1.905484455526863 | 2.011823695557204 | 1.617446131862227 | 2.34880705589965 |
| GW0742 5uM | 2.995847383076792 | 1.937855580725823 | 2.531204788110067 | 2.429306296455538 | 5.17101959782904 |
| GW0742 10uM | 4.826337221390466 | 4.034009033837352 | 6.637672924054463 | 2.596401197588217 | 3.244422681249707 |
| GW501516 1uM | 1.503786701931866 | 1.530775701889196 | 1.31082778995864 | 1.425416760647395 | 1.15057517053764 |
| GW501516 5uM | 1.754128449899367 | 1.664291408509541 | 3.208859294185678 | 2.560169060385727 | 3.821249428200716 |
| GW501516 10.uM | 2.494436345943204 | 2.919395330167774 | 3.819191084919017 | 3.417186835573553 | 5.255558196861048 |
| L165 1uM | 1.076726802742131 | 1.616492045759867 | 1.335359674944703 | 1.699101218751921 | 1.384648119025494 |
| L165 5uM | 2.534614589857447 | 2.382842075138824 | 2.567213279807114 | 3.502959339950518 | 2.892106522817841 |
| L165 10uM | 2.655658159355837 | 2.19312446530728 | 3.370873487475196 | 4.061724603716478 | 4.85351727865278 |*
*
*
*
*
*
*
*
*
*
*
*
*
*
*
*
*
*
*
*
*
*
*
*
*
Ctrl
GW0742
GW501516
L-165
Relative expression (fold change)
1 5 10
1 5 10
1 5 10 µM
VIM
ZEB1
SLUG
SNAIL
LOXL2
Ctrl
GW0742
GW501516
L-165
B
D
NT
sh#1 PPARD
sh#2 PPARD
sh#3 PPARD
### Chart
| Category | NT | 900 | 901 | 902 |
|---|---|---|---|---|
| Cont | 1.0281352875738932 | 0.6905256098943608 | 1.0126158988564353 | 1.5823887416953804 |
| MCM | 3.5522579397423266 | 1.1702386158415095 | 1.196174645287955 | 1.1175346226675305 |
| Eto | 3.5518682029033095 | 0.7193238715376601 | 1.25509470815575 | 1.4824951281073615 |
| L-165 | 3.1129250491126665 | 0.8840869786483703 | 0.9975570166749833 | 1.5308349845616245 |
### Chart
| Category | NT | 900 | 901 | 902 |
|---|---|---|---|---|
| Cont | 1.017305500763657 | 0.9668689709843954 | 1.0549599415337774 | 0.9763016959851457 |
| MCM | 3.0012949718368467 | 1.3531279951447894 | 1.1052057399342998 | 1.8743593336437436 |
| Eto | 2.3081310064667186 | 1.269989464446706 | 1.4964410637032843 | 1.4877803326213845 |
| L-165 | 5.589425366520307 | 1.3907443175536895 | 1.9246751369523791 | 2.0136776981666866 |
### Chart
| Category | NT | 900 | 901 | 902 |
|---|---|---|---|---|
| Cont | 1.0102500184448553 | 0.452763076775939 | 0.563903817476339 | 0.46378817809688255 |
| MCM | 1.8864097694412783 | 0.4999421214042243 | 0.7277885651819267 | 0.9023791553886852 |
| Eto | 2.823524879290245 | 1.347627332823989 | 1.0790789885243435 | 1.2870524235898144 |
| L-165 | 3.441538217156495 | 0.7827672580584949 | 0.8811455027271667 | 1.0614744410953345 |**
**
**
***
**
***
PPARD relative expression
(fold change)
##
***
##
###
**
##
###
###
#
###
#
**
#
###
PDAC-215
PDAC-253
PDAC-354
