# Supplementary material for: PPARδ Orchestrates a Prometastatic Metabolic Response to Microenvironmental Cues in Pancreatic Cancer
Source: Cancer Res. 2025 Jul 3;85(17):3275–91. doi: 10.1158/0008-5472.CAN-24-3475 (PMC12402788; doi:10.1158/0008-5472.CAN-24-3475)
Supplement: Figure S9 — Effects of PPARD knockdown in the expression of genes related with the EMT program in different PDAC PDXs [file can-24-3475_figure_s9_suppsf9.pptx]

## Slide 1
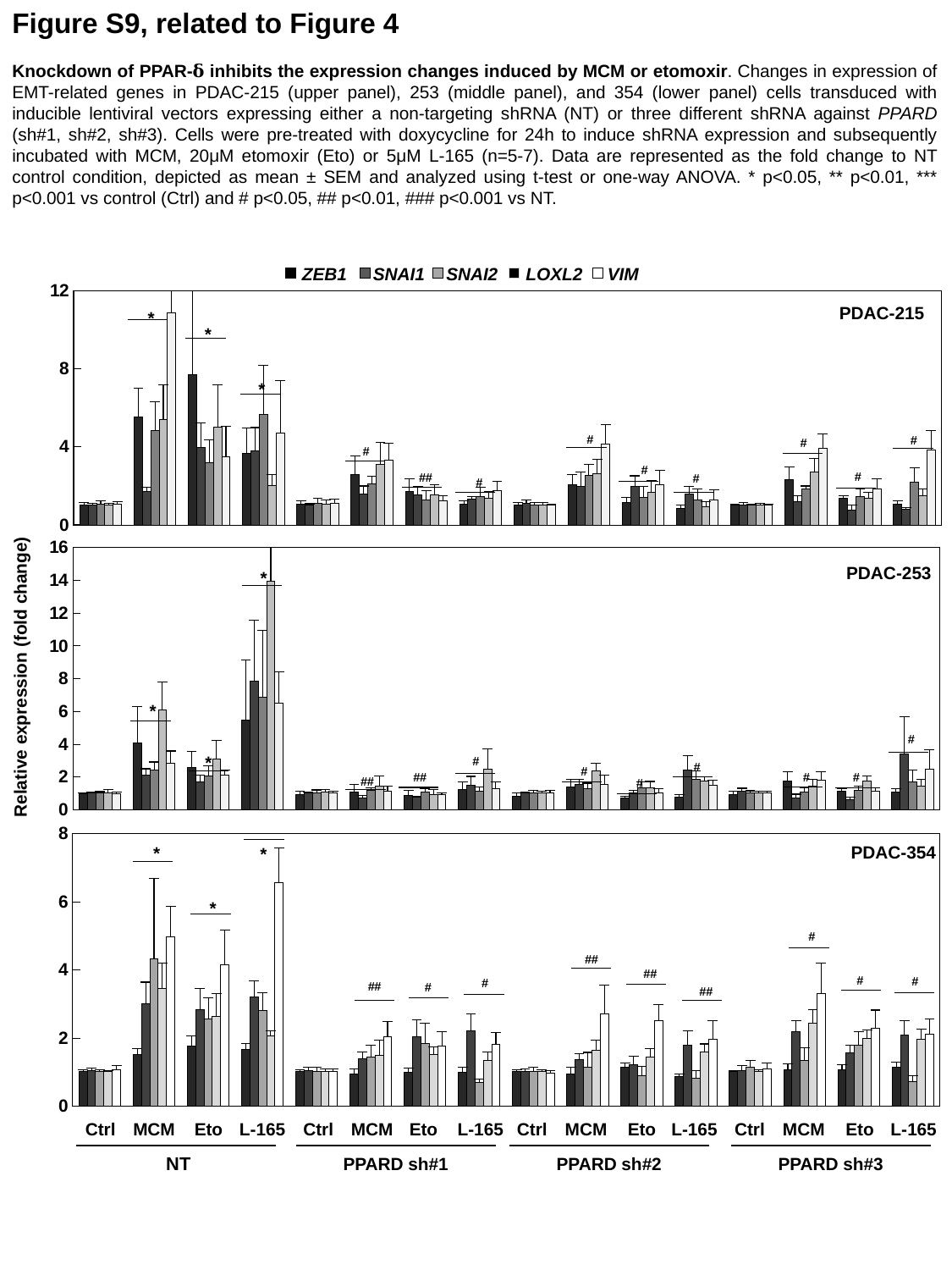

Figure S9, related to Figure 4
Knockdown of PPAR-𝛅 inhibits the expression changes induced by MCM or etomoxir. Changes in expression of EMT-related genes in PDAC-215 (upper panel), 253 (middle panel), and 354 (lower panel) cells transduced with inducible lentiviral vectors expressing either a non-targeting shRNA (NT) or three different shRNA against PPARD (sh#1, sh#2, sh#3). Cells were pre-treated with doxycycline for 24h to induce shRNA expression and subsequently incubated with MCM, 20μM etomoxir (Eto) or 5μM L-165 (n=5-7). Data are represented as the fold change to NT control condition, depicted as mean ± SEM and analyzed using t-test or one-way ANOVA. * p<0.05, ** p<0.01, *** p<0.001 vs control (Ctrl) and # p<0.05, ## p<0.01, ### p<0.001 vs NT.
VIM
SNAI2
LOXL2
ZEB1
SNAI1
### Chart
| Category | VIM | SNAIL | SLUG | ZEB1 | LOXL2 |
|---|---|---|---|---|---|
| Cont | 1.034749588033547 | 1.02252639952032 | 1.064669912043573 | 1.015816657224756 | 1.04565303275683 |
| MCM | 5.533478209872815 | 1.713366303794692 | 4.820867272470874 | 5.396507549437866 | 10.87861398710692 |
| Eto | 7.712976936928256 | 3.962277152011229 | 3.170787408429413 | 5.000680685048338 | 3.513354466881751 |
| L-165 | 3.655459084517254 | 3.798841792269476 | 5.687298662455821 | 2.023276049955171 | 4.711790337871402 |
| Cont | 1.05731036915601 | 1.011445513189535 | 1.116857483844131 | 1.080271353000126 | 1.087864366058003 |
| MCM | 2.567555303788612 | 1.600002916832708 | 2.112619750935352 | 3.122582554991261 | 3.317444873220034 |
| Eto | 1.694455388160678 | 1.556988854886705 | 1.301112303580225 | 1.542331751298639 | 1.248312504073943 |
| L-165 | 1.04330399075711 | 1.324780885094474 | 1.454684052286359 | 1.368874196699882 | 1.751840542438614 |
| Cont | 1.02599088958191 | 1.127089256855462 | 1.035678022929302 | 1.035276903685926 | 1.007705493381555 |
| MCM | 2.045506824579747 | 1.960263852434394 | 2.544299452094953 | 2.616636745922865 | 4.126707325502185 |
| Eto | 1.148035949417734 | 1.964970657090044 | 1.422337061421757 | 1.673014820747576 | 2.054142095524607 |
| L-165 | 0.839942369780705 | 1.582550680363885 | 1.301170294531758 | 0.940774565331895 | 1.281865088142506 |
| Cont | 1.004789407317668 | 1.028880905442246 | 1.011450544403341 | 1.022485324117973 | 1.007397139019689 |
| MCM | 2.339852940522328 | 1.191038697940827 | 1.832114816196391 | 2.711588935433836 | 3.939121592333333 |
| Eto | 1.348511860009667 | 0.779822544431982 | 1.476059784060203 | 1.386870126815695 | 1.85063365361688 |
| L-165 | 1.043224679256464 | 0.790118849308746 | 2.200061812944848 | 1.494515410766027 | 3.836080726580294 |PDAC-215
*
*
*
#
#
#
#
#
#
##
#
#
### Chart
| Category | VIM | SNAIL | SLUG | ZEB1 | LOXL2 |
|---|---|---|---|---|---|
| Cont | 1.002313664185245 | 1.014107135686602 | 1.016959356685261 | 1.052428486472947 | 1.005890180475982 |
| MCM | 4.094694359451005 | 2.131810491699414 | 2.444745289572324 | 6.069236256383506 | 2.854005082352753 |
| Eto | 2.601997311165434 | 1.696148825007716 | 2.087202539826424 | 3.120106920410992 | 2.134951099463152 |
| L-165 | 5.454182286820886 | 7.847675417089039 | 6.861558406638395 | 13.9223081714059 | 6.518650163002946 |
| Cont | 0.938283371785544 | 1.013345071146257 | 1.044049953716873 | 1.062473046111055 | 1.027154183964648 |
| MCM | 1.099525716526107 | 0.706178491341689 | 1.209884568419951 | 1.464851437388238 | 1.14675037785968 |
| Eto | 0.890862510807666 | 0.761347772826241 | 1.106325230387912 | 0.932822549296778 | 0.924045854755656 |
| L-165 | 1.238905903620135 | 1.510578420581871 | 1.123334175765379 | 2.496176526800997 | 1.270343700295738 |
| Cont | 0.811615756891391 | 1.01289785793655 | 1.038130754340636 | 1.01787346650445 | 1.03869199463178 |
| MCM | 1.377935461139813 | 1.529831268835244 | 1.28416550231942 | 2.3730177209005 | 1.558150906305363 |
| Eto | 0.70383154938523 | 1.045088701650888 | 1.355830062143153 | 1.350694181868443 | 1.039078945680321 |
| L-165 | 0.795939632085372 | 2.434516567122308 | 1.861997147313732 | 1.74710386877169 | 1.502964854960754 |
| Cont | 0.936478602864353 | 1.141877067039097 | 1.027050361412547 | 1.02388572417921 | 1.026376965993646 |
| MCM | 1.74252106580096 | 0.727701360898818 | 1.094376275662603 | 1.419394631619 | 1.81215932150926 |
| Eto | 1.155553619815222 | 0.608106321284907 | 1.208608709238884 | 1.75940380687284 | 1.11643492720389 |
| L-165 | 1.085496365679036 | 3.421105477314608 | 1.712471914815723 | 1.45909193930856 | 2.456114601625792 |PDAC-253
*
Relative expression (fold change)
*
#
*
#
#
#
##
#
#
##
#
### Chart
| Category | VIM | SLUG | SNAIL | ZEB1 | LOXL2 |
|---|---|---|---|---|---|
| Cont | 1.013574703577178 | 1.03486535345058 | 1.024451059762075 | 1.007102207435538 | 1.060564223751187 |
| MCM | 1.518643255489438 | 2.996888732023946 | 4.33726836153958 | 3.467517976124247 | 4.97215834808567 |
| Eto | 1.772292304354233 | 2.824825705413049 | 2.558790575359428 | 2.639171824341065 | 4.155260311039678 |
| L-165 | 1.670217399904247 | 3.205084751763558 | 2.803229550707035 | 2.071978944391903 | 6.563003173883467 |
| Cont | 1.015306926352902 | 1.041967376722424 | 1.02324840499908 | 1.019193661472345 | 1.019826672904169 |
| MCM | 0.95372854804633 | 1.392184731260333 | 1.443321922383394 | 1.500672385966338 | 2.035255767620299 |
| Eto | 0.999494996413904 | 2.026036534115057 | 1.845088047243683 | 1.526439826012392 | 1.757728029502932 |
| L-165 | 1.000630458663494 | 2.22405661260313 | 0.706341011476487 | 1.353228744479539 | 1.808565460325406 |
| Cont | 1.013801107216016 | 1.016647424957865 | 1.028500540967581 | 1.013803128648668 | 0.970320723939607 |
| MCM | 0.953081046947517 | 1.3594249281697 | 1.138985396525888 | 1.642994591210971 | 2.716732215467801 |
| Eto | 1.134034793118837 | 1.205701697122767 | 0.901764258590912 | 1.437247104147167 | 2.508300381799863 |
| L-165 | 0.876826129649964 | 1.79897164731757 | 0.831240083011958 | 1.58651733904413 | 1.969101402826368 |
| Cont | 1.008160631595767 | 1.054909498009978 | 1.134732845186988 | 1.009941006719127 | 1.10399294603924 |
| MCM | 1.067116483353933 | 2.196895170447124 | 1.335304890994525 | 2.425541022249634 | 3.309653284351912 |
| Eto | 1.08056165319909 | 1.554838212955336 | 1.79468261422652 | 1.984947105986834 | 2.28795766629207 |
| L-165 | 1.147630844825097 | 2.087552858950595 | 0.727181347617343 | 1.970889990229981 | 2.122891955430873 |PDAC-354
*
*
*
#
##
##
#
#
#
#
##
Ctrl
MCM
Eto
L-165
Ctrl
MCM
Eto
L-165
Ctrl
MCM
Eto
L-165
Ctrl
MCM
Eto
L-165
NT
PPARD sh#1
PPARD sh#2
PPARD sh#3
