# Supplementary material for: PPARδ Orchestrates a Prometastatic Metabolic Response to Microenvironmental Cues in Pancreatic Cancer
Source: Cancer Res. 2025 Jul 3;85(17):3275–91. doi: 10.1158/0008-5472.CAN-24-3475 (PMC12402788; doi:10.1158/0008-5472.CAN-24-3475)
Supplement: Figure S10 — The EMT program is related to MYC upregulation and PGC1A downregulation [file can-24-3475_figure_s10_suppsf10.pptx]

## Slide 1
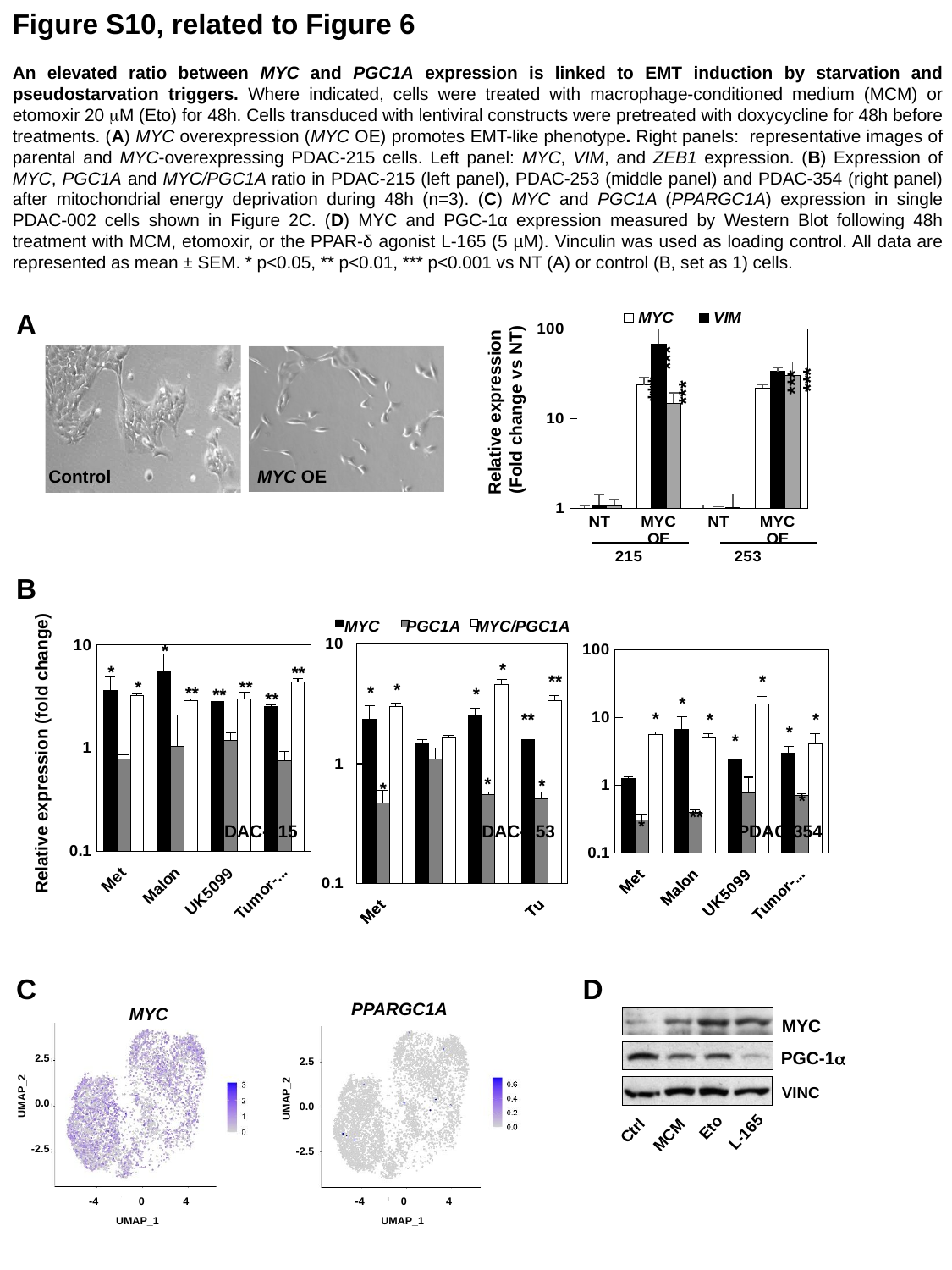

Figure S10, related to Figure 6
An elevated ratio between MYC and PGC1A expression is linked to EMT induction by starvation and pseudostarvation triggers. Where indicated, cells were treated with macrophage-conditioned medium (MCM) or etomoxir 20 mM (Eto) for 48h. Cells transduced with lentiviral constructs were pretreated with doxycycline for 48h before treatments. (A) MYC overexpression (MYC OE) promotes EMT-like phenotype. Right panels: representative images of parental and MYC-overexpressing PDAC-215 cells. Left panel: MYC, VIM, and ZEB1 expression. (B) Expression of MYC, PGC1A and MYC/PGC1A ratio in PDAC-215 (left panel), PDAC-253 (middle panel) and PDAC-354 (right panel) after mitochondrial energy deprivation during 48h (n=3). (C) MYC and PGC1A (PPARGC1A) expression in single PDAC-002 cells shown in Figure 2C. (D) MYC and PGC-1α expression measured by Western Blot following 48h treatment with MCM, etomoxir, or the PPAR-δ agonist L-165 (5 µM). Vinculin was used as loading control. All data are represented as mean ± SEM. * p<0.05, ** p<0.01, *** p<0.001 vs NT (A) or control (B, set as 1) cells.
A
### Chart
| Category | MYC | VIM | ZEB1 |
|---|---|---|---|
| NT | 1.0033787073365 | 1.09474540458184 | 1.071006449836846 |
| MYC OE | 23.56058427284418 | 67.60081982672202 | 14.84304499868734 |
| NT | 1.001539929048073 | 1.001510952193592 | 1.019253005997857 |
| MYC OE | 21.8507630595347 | 33.50718794826571 | 30.02955435098209 |***
***
***
***
***
***
Relative expression
(Fold change vs NT)
Control
MYC OE
B
MYC
PGC1A
MYC/PGC1A
### Chart
| Category | MYC | PGC1A | |
|---|---|---|---|
| Met | 1.257270461345201 | 0.3037323662001308 | 5.522174 |
| Malon | 6.57952426725686 | 0.3935148800513109 | 4.960367892690134 |
| UK5099 | 2.3836882788959497 | 0.764349661737409 | 15.662378598444645 |
| Tumor-like | 2.9941460989457314 | 0.7074012690161234 | 4.11537177978953 |*
### Chart
| Category | MYC | PGC1A | |
|---|---|---|---|
| Met | 2.342959284839429 | 0.4715012268664359 | 2.98875 |
| Malon | 1.475908280817495 | 1.100517834988564 | 1.6294494174159724 |
| UK5099 | 2.5422656603144262 | 0.553747700158511 | 4.541971215958086 |
| Tumor-like | 1.5888686937617567 | 0.507480112147164 | 3.373722658656507 |
### Chart
| Category | | PGC1A | |
|---|---|---|---|
| Met | 3.630916519236785 | 0.7842212729210842 | 3.21167 |
| Malon | 5.546304299832815 | 1.03504032743843 | 2.8779071854887452 |
| UK5099 | 2.8490641983464684 | 1.1865983511226994 | 3.0199982111550066 |
| Tumor-like | 2.553624025758321 | 0.7585670087328925 | 4.381762110861335 |*
*
**
*
**
**
*
*
*
**
*
**
**
*
*
**
*
*
*
*
Relative expression (fold change)
*
*
*
*
**
*
PDAC-215
PDAC-253
PDAC-354
C
D
PPARGC1A
MYC
MYC
PGC-1a
VINC
Eto
Ctrl
L-165
MCM
2.5
2.5
UMAP_2
UMAP_2
0.0
0.0
-2.5
-2.5
-4
0
4
-4
0
4
UMAP_1
UMAP_1
