# Supplementary material for: PPARδ Orchestrates a Prometastatic Metabolic Response to Microenvironmental Cues in Pancreatic Cancer
Source: Cancer Res. 2025 Jul 3;85(17):3275–91. doi: 10.1158/0008-5472.CAN-24-3475 (PMC12402788; doi:10.1158/0008-5472.CAN-24-3475)
Supplement: Figure S11 — PPARD knockdown modifies the expression ratio between MYC and PGC1A [file can-24-3475_figure_s11_suppsf11.pptx]

## Slide 1
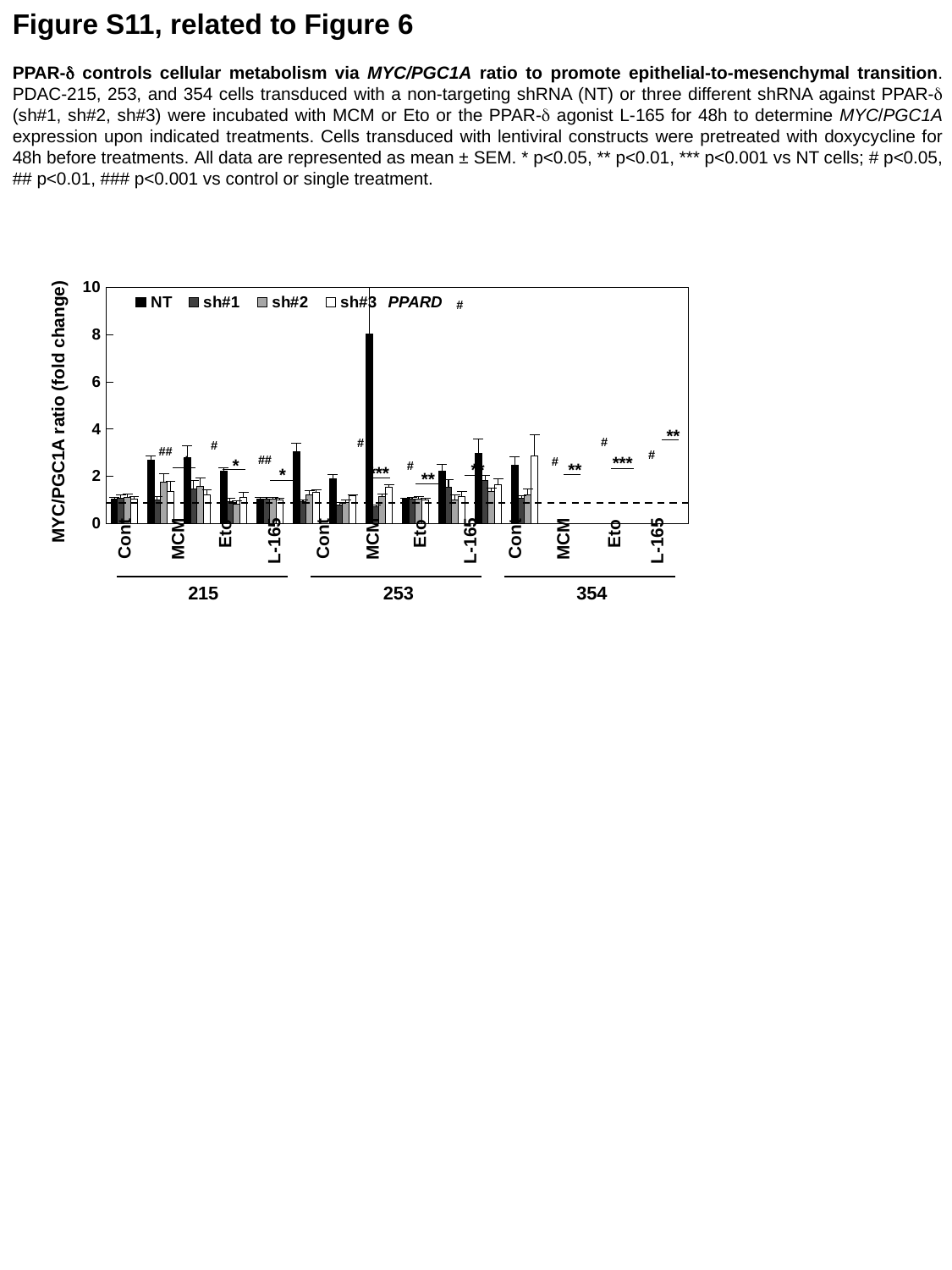

Figure S11, related to Figure 6
PPAR-d controls cellular metabolism via MYC/PGC1A ratio to promote epithelial-to-mesenchymal transition. PDAC-215, 253, and 354 cells transduced with a non-targeting shRNA (NT) or three different shRNA against PPAR-d (sh#1, sh#2, sh#3) were incubated with MCM or Eto or the PPAR-d agonist L-165 for 48h to determine MYC/PGC1A expression upon indicated treatments. Cells transduced with lentiviral constructs were pretreated with doxycycline for 48h before treatments. All data are represented as mean ± SEM. * p<0.05, ** p<0.01, *** p<0.001 vs NT cells; # p<0.05, ## p<0.01, ### p<0.001 vs control or single treatment.
[unsupported chart]
PPARD
#
MYC/PGC1A ratio (fold change)
**
#
#
#
##
#
*
***
##
#
*
**
**
#
***
*
**
Eto
Eto
Eto
Cont
Cont
Cont
MCM
MCM
MCM
L-165
L-165
L-165
215
253
354
