# Supplementary material for: PPARδ Orchestrates a Prometastatic Metabolic Response to Microenvironmental Cues in Pancreatic Cancer
Source: Cancer Res. 2025 Jul 3;85(17):3275–91. doi: 10.1158/0008-5472.CAN-24-3475 (PMC12402788; doi:10.1158/0008-5472.CAN-24-3475)
Supplement: Figure S12 — MYC downregulation reverses the prometastatic phenotype induced by etomoxir and MCM [file can-24-3475_figure_s12_suppsf12.pptx]

## Slide 1
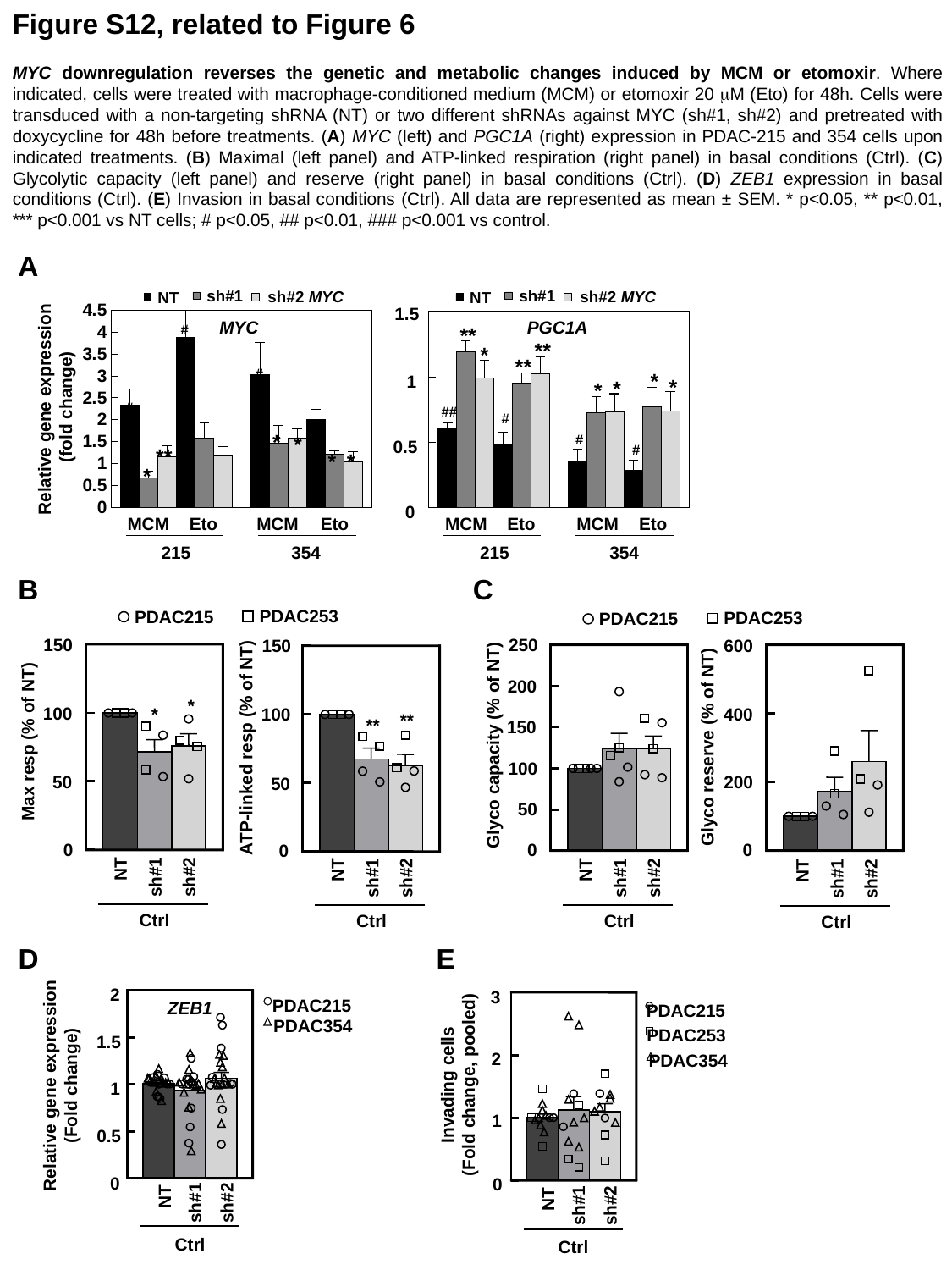

Figure S12, related to Figure 6
MYC downregulation reverses the genetic and metabolic changes induced by MCM or etomoxir. Where indicated, cells were treated with macrophage-conditioned medium (MCM) or etomoxir 20 mM (Eto) for 48h. Cells were transduced with a non-targeting shRNA (NT) or two different shRNAs against MYC (sh#1, sh#2) and pretreated with doxycycline for 48h before treatments. (A) MYC (left) and PGC1A (right) expression in PDAC-215 and 354 cells upon indicated treatments. (B) Maximal (left panel) and ATP-linked respiration (right panel) in basal conditions (Ctrl). (C) Glycolytic capacity (left panel) and reserve (right panel) in basal conditions (Ctrl). (D) ZEB1 expression in basal conditions (Ctrl). (E) Invasion in basal conditions (Ctrl). All data are represented as mean ± SEM. * p<0.05, ** p<0.01, *** p<0.001 vs NT cells; # p<0.05, ## p<0.01, ### p<0.001 vs control.
A
### Chart
| Category | | | | | | |
|---|---|---|---|---|---|---|
| MCM | 0.608121392367512 | 1.190830813681744 | 0.991373567631455 | 0.47797799418059 | 0.948682704152105 | 1.022040031798656 |
| Eto | 0.3503050509134 | 0.723381196296438 | 0.733783384756206 | 0.283698720798324 | 0.770180231841649 | 0.73899188597683 |sh#1
sh#2 MYC
NT
sh#1
sh#2 MYC
NT
### Chart
| Category | | | | | | |
|---|---|---|---|---|---|---|
| MCM | 2.338847963083432 | 0.671478568824174 | 1.16257212365025 | 3.875098710621049 | 1.587951613741104 | 1.19577299307296 |
| Eto | 3.021242066659068 | 1.471836306729805 | 1.583333372355427 | 1.998265772285618 | 1.20445055622801 | 1.030292326434983 |1.5
MYC
PGC1A
#
**
**
*
**
#
*
1
*
*
*
Relative gene expression
(fold change)
#
##
#
#
*
#
*
0.5
#
**
*
*
*
*
0
MCM
Eto
MCM
Eto
MCM
Eto
MCM
Eto
215
354
215
354
B
C
PDAC253
PDAC215
150
150
*
100
100
*
**
**
Max resp (% of NT)
ATP-linked resp (% of NT)
50
50
0
0
NT
NT
sh#1
sh#2
sh#1
sh#2
Ctrl
Ctrl
PDAC253
PDAC215
250
600
200
400
150
Glyco capacity (% of NT)
Glyco reserve (% of NT)
100
200
50
0
0
NT
NT
sh#1
sh#2
sh#1
sh#2
Ctrl
Ctrl
D
E
2
PDAC215
ZEB1
PDAC354
1.5
Relative gene expression
(Fold change)
1
0.5
0
NT
sh#1
sh#2
Ctrl
3
PDAC215
PDAC253
2
PDAC354
Invading cells
(Fold change, pooled)
1
0
NT
sh#1
sh#2
Ctrl
