# Supplementary material for: PPARδ Orchestrates a Prometastatic Metabolic Response to Microenvironmental Cues in Pancreatic Cancer
Source: Cancer Res. 2025 Jul 3;85(17):3275–91. doi: 10.1158/0008-5472.CAN-24-3475 (PMC12402788; doi:10.1158/0008-5472.CAN-24-3475)
Supplement: Figure S13 — Effects of MYC inhibition with mycro3 on EMT genes expression and changes induced by PGC1A overexpression at the functional level [file can-24-3475_figure_s13_suppsf13.pptx]

## Slide 1
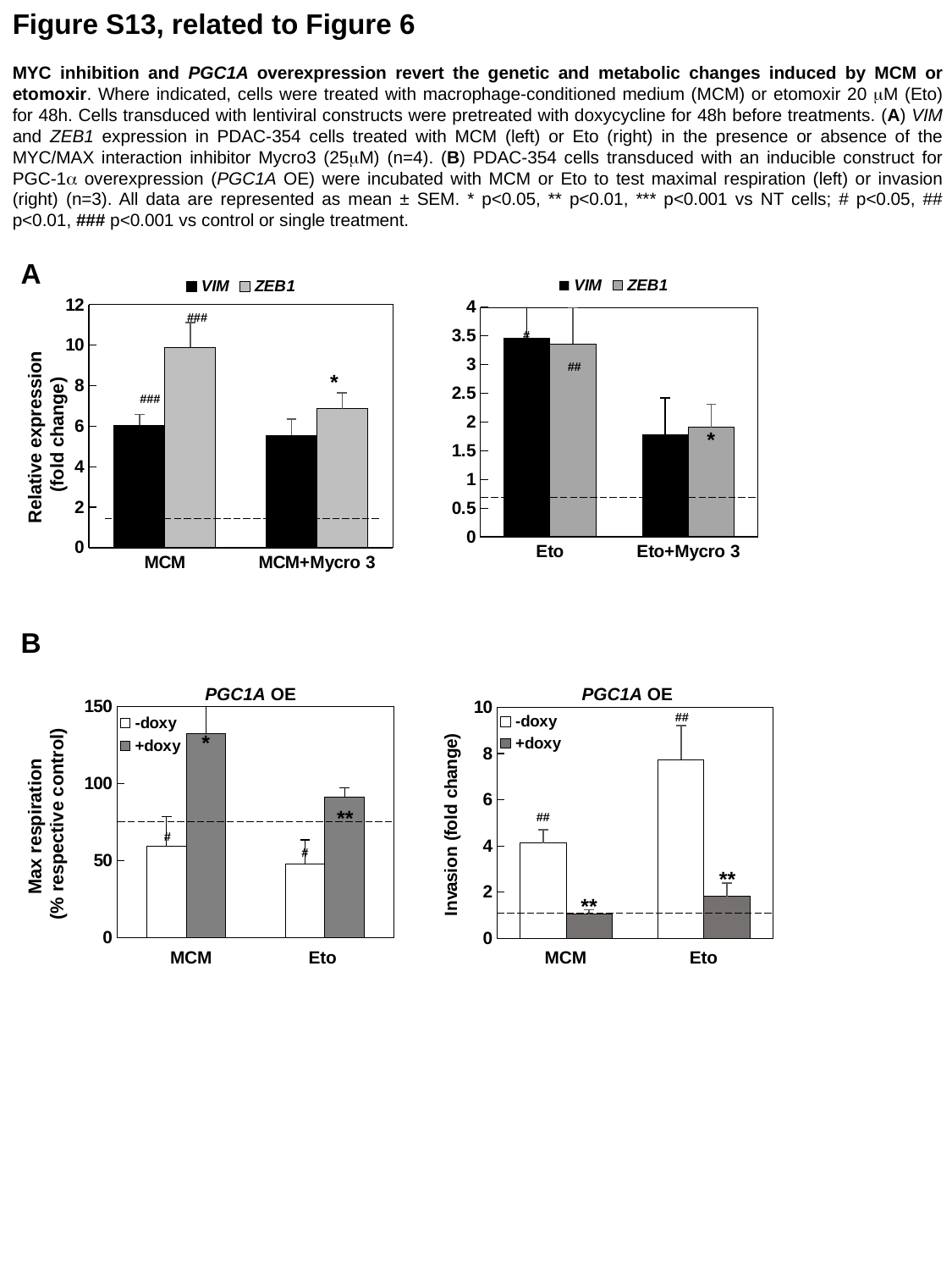

Figure S13, related to Figure 6
MYC inhibition and PGC1A overexpression revert the genetic and metabolic changes induced by MCM or etomoxir. Where indicated, cells were treated with macrophage-conditioned medium (MCM) or etomoxir 20 mM (Eto) for 48h. Cells transduced with lentiviral constructs were pretreated with doxycycline for 48h before treatments. (A) VIM and ZEB1 expression in PDAC-354 cells treated with MCM (left) or Eto (right) in the presence or absence of the MYC/MAX interaction inhibitor Mycro3 (25mM) (n=4). (B) PDAC-354 cells transduced with an inducible construct for PGC-1a overexpression (PGC1A OE) were incubated with MCM or Eto to test maximal respiration (left) or invasion (right) (n=3). All data are represented as mean ± SEM. * p<0.05, ** p<0.01, *** p<0.001 vs NT cells; # p<0.05, ## p<0.01, ### p<0.001 vs control or single treatment.
A
### Chart
| Category | VIM | ZEB1 |
|---|---|---|
| MCM | 6.051931298876686 | 9.90295054423059 |
| MCM+Mycro 3 | 5.537849262956308 | 6.86898894936809 |
### Chart
| Category | VIM | ZEB1 |
|---|---|---|
| Eto | 3.471676134328741 | 3.363138598659584 |
| Eto+Mycro 3 | 1.788968778161371 | 1.904029685462145 |###
#
##
*
###
Relative expression
(fold change)
*
### Chart
| Category | | |
|---|---|---|*
**
#
#
### Chart
| Category | | |
|---|---|---|
| MCM | 4.121428571428567 | 1.038654970760234 |
| eto 20 | 7.73095238095238 | 1.813684210526316 |##
##
**
**
PGC1A OE
PGC1A OE
Max respiration
(% respective control)
MCM
Eto
MCM
Eto
B
