# Supplementary material for: PPARδ Orchestrates a Prometastatic Metabolic Response to Microenvironmental Cues in Pancreatic Cancer
Source: Cancer Res. 2025 Jul 3;85(17):3275–91. doi: 10.1158/0008-5472.CAN-24-3475 (PMC12402788; doi:10.1158/0008-5472.CAN-24-3475)
Supplement: Figure S14 — Effects of pharmacological modulation of PPAR-δ on invasion and metastasis in vivo, using the highly metastatic PDX CTCA [file can-24-3475_figure_s14_suppsf14.pptx]

## Slide 1
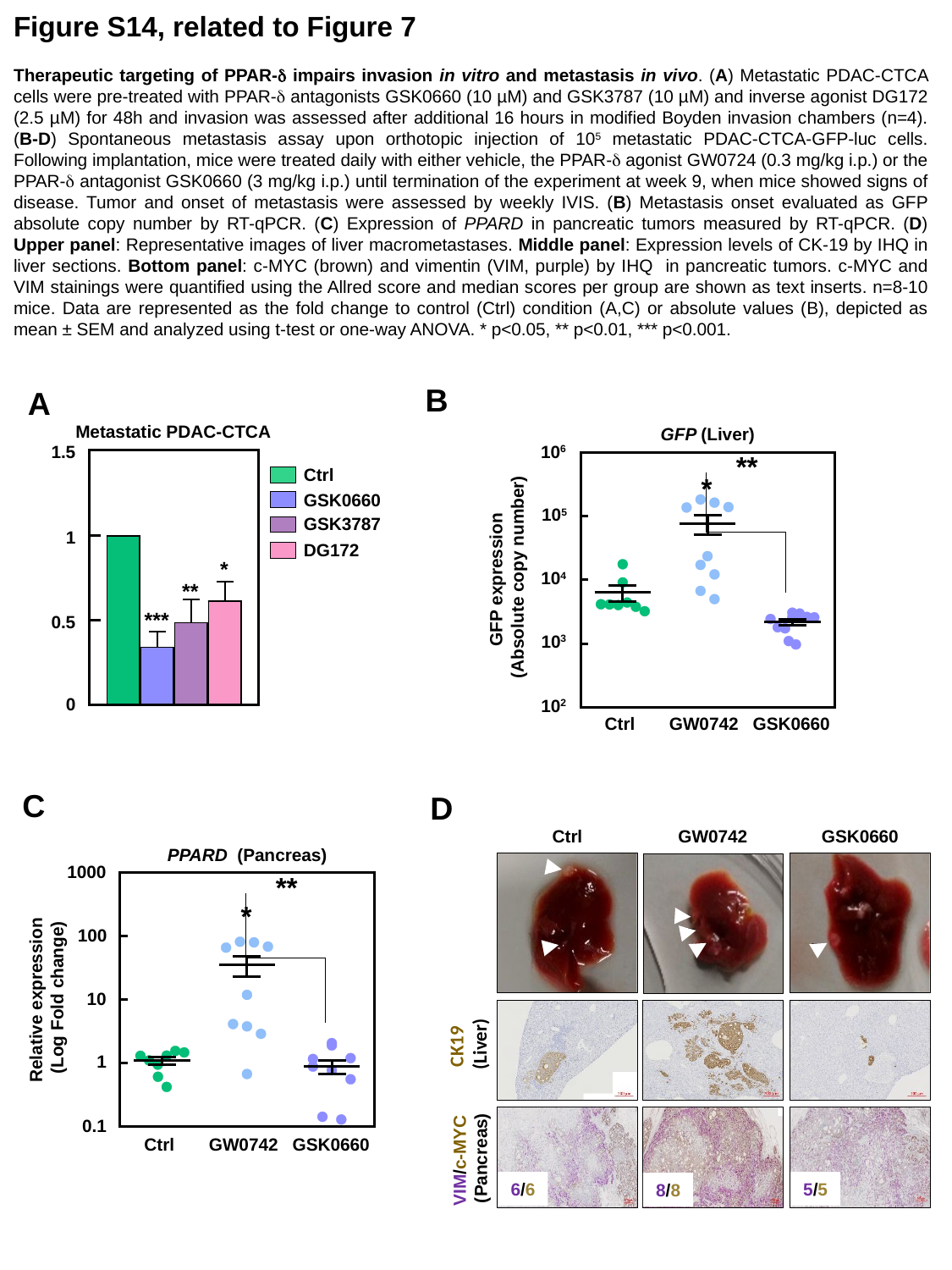

Figure S14, related to Figure 7
Therapeutic targeting of PPAR-d impairs invasion in vitro and metastasis in vivo. (A) Metastatic PDAC-CTCA cells were pre-treated with PPAR-d antagonists GSK0660 (10 µM) and GSK3787 (10 µM) and inverse agonist DG172 (2.5 µM) for 48h and invasion was assessed after additional 16 hours in modified Boyden invasion chambers (n=4). (B-D) Spontaneous metastasis assay upon orthotopic injection of 105 metastatic PDAC-CTCA-GFP-luc cells. Following implantation, mice were treated daily with either vehicle, the PPAR-d agonist GW0724 (0.3 mg/kg i.p.) or the PPAR-d antagonist GSK0660 (3 mg/kg i.p.) until termination of the experiment at week 9, when mice showed signs of disease. Tumor and onset of metastasis were assessed by weekly IVIS. (B) Metastasis onset evaluated as GFP absolute copy number by RT-qPCR. (C) Expression of PPARD in pancreatic tumors measured by RT-qPCR. (D) Upper panel: Representative images of liver macrometastases. Middle panel: Expression levels of CK-19 by IHQ in liver sections. Bottom panel: c-MYC (brown) and vimentin (VIM, purple) by IHQ in pancreatic tumors. c-MYC and VIM stainings were quantified using the Allred score and median scores per group are shown as text inserts. n=8-10 mice. Data are represented as the fold change to control (Ctrl) condition (A,C) or absolute values (B), depicted as mean ± SEM and analyzed using t-test or one-way ANOVA. * p<0.05, ** p<0.01, *** p<0.001.
B
A
Metastatic PDAC-CTCA
1.5
1
**
***
0.5
0
Ctrl
GSK0660
GSK3787
DG172
*
GFP (Liver)
106
105
GFP expression
(Absolute copy number)
104
103
102
Ctrl
GW0742
GSK0660
**
*
C
D
Ctrl
GW0742
GSK0660
CK19
(Liver)
VIM/c-MYC
(Pancreas)
6/6
5/5
8/8
PPARD (Pancreas)
1000
100
Relative expression
(Log Fold change)
10
1
0.1
Ctrl
GW0742
GSK0660
**
*
